# Expression of the Arabidopsis Mg-chelatase H subunit alleviates iron deficiency-induced stress in transgenic rice

**DOI:** 10.3389/fpls.2023.1098808

**Published:** 2023-03-02

**Authors:** Lien Hong Tran, Jin-Gil Kim, Sunyo Jung

**Affiliations:** School of Life Sciences, BK21 FOUR KNU Creative BioResearch Group, Kyungpook National University, Daegu, Republic of Korea

**Keywords:** *AtCHLH*, Fe deficiency, Fe homeostasis, porphyrin biosynthesis, transgenic rice

## Abstract

The most common symptom of iron (Fe) deficiency in plants is leaf chlorosis caused by impairment of chlorophyll biosynthesis. Magnesium (Mg)-chelatase H subunit (CHLH) is a key component in both chlorophyll biosynthesis and plastid signaling, but its role in Fe deficiency is poorly understood. Heterologous expression of the *Arabidopsis thaliana* Mg-chelatase H subunit gene (*AtCHLH*) increased Mg-chelatase activity by up to 6-fold and abundance of its product, Mg-protoporphyrin IX (Mg-Proto IX), by 60–75% in transgenic rice (*Oryza sativa*) seedlings compared to wild-type (WT) controls. Noticeably, the transgenic seedlings showed alleviation of Fe deficiency symptoms, as evidenced by their less pronounced leaf chlorosis and lower declines in shoot growth, chlorophyll contents, and photosynthetic efficiency, as indicated by *F*_v_/*F*_m_ and electron transport rate, compared to those in WT seedlings under Fe deficiency. Porphyrin metabolism was differentially regulated by Fe deficiency between WT and transgenic seedlings, particularly with a higher level of Mg-Proto IX in transgenic lines, showing that overexpression of *AtCHLH* reprograms porphyrin metabolism in transgenic rice. Leaves of Fe-deficient transgenic seedlings exhibited greater upregulation of deoxymugineic acid biosynthesis-related genes (i.e., *NAS*, *NAS2*, and *NAAT1*), *YSL2* transporter gene, and Fe-related transcription factor genes *IRO2* and *IDEF2* than those of WT, which may also partly contribute to alleviating Fe deficiency. Although *At*CHLH was postulated to act as a receptor for abscisic acid (ABA), exogenous ABA did not alter the phenotypes of Fe-deficient WT or transgenic seedlings. Our study demonstrates that modulation of porphyrin biosynthesis through expression of *AtCHLH* in transgenic rice alleviates Fe deficiency-induced stress, suggesting a possible role for CHLH in Fe deficiency responses.

## Introduction

Iron (Fe) is an essential microelement for all organisms as a protein cofactor in cellular processes including chlorophyll biosynthesis, photosynthetic electron transport, scavenging of reactive oxygen species (ROS), and respiration (Spiller et al., 1982; Eide et al., 1996; Kobayashi et al., 2019; Kroh and Pilon, 2020). Because Fe is commonly limiting in the environment, plants must acclimate to Fe availability and avoid the effects of Fe deficiency (Eide et al., 1996; Vert et al., 2002; Kroh and Pilon, 2020). Graminaceous plants use a chelation-based mechanism (strategy II) and release phytosiderophores of the mugineic acid (MA) family from their roots to chelate Fe^3+^ in the soil (Kobayashi and Nishizawa, 2012). Fe^3+^-MAs are then taken up through yellow stripe-like (YSL) transporters that play a crucial role in Fe homeostasis (Inoue et al., 2009; Ishimaru et al., 2010). In rice (*Oryza sativa*), the synthesis of MAs is mediated by a series of enzymes, including nicotianamine synthase (NAS), nicotianamine aminotransferase (NAAT), and deoxymugineic acid (DMA) synthase (Kobayashi et al., 2014). Iron-related basic helix-loop-helix-type (bHLH) transcription factor 2 (*Os*IRO2) positively regulates various Fe deficiency-induced genes related to DMA-based Fe acquisition, including *OsNAS1*, *OsNAS2*, *OsNAAT1*, and *OsYSL15* (Ogo et al., 2007). Iron deficiency-responsive element binding factor 1 (IDEF1) regulates most genes known to be involved in Fe(III)-DMA and Fe^2+^ uptake, as well as Fe translocation, while IDEF2 regulates *OsYSL2* and other Fe deficiency-inducible genes which might be involved in Fe translocation (Kobayashi et al., 2014). Rice is unusual among the graminaceous plants in that it also borrows uptake mechanisms from strategy I, such as taking up Fe^2+^ using an iron-regulated transporter 1 (IRT1)-type transporter (Kobayashi and Nishizawa, 2012).

Chlorosis, a typical symptom of Fe deficiency, is caused by impaired chlorophyll biosynthesis and is associated with decreased photosynthetic rates (Terry, 1980) because the photosynthetic electron transport chain has a high Fe quota for the many proteins containing heme and Fe–sulfur (S) clusters (Kroh and Pilon, 2020). Enzymes involved in chlorophyll metabolism, including magnesium-protoporphyrin IX monomethyl ester (Mg-Proto IX ME) cyclase and chlorophyllide A oxygenase, are Fe–S cluster-dependent enzymes (Kroh and Pilon, 2020). Chlorophyll biosynthesis starts with glutamyl-tRNA^Glu^, which is converted to 5-aminolevulinic acid (ALA), which itself is metabolized to form tetrapyrroles (Beale and Weinstein, 1990). The last common step of the porphyrin biosynthesis pathway is the oxidation of protoporphyrinogen IX into Proto IX by protoporphyrinogen oxidase (PPO) before the pathway branches to produce chlorophyll or heme. Mg-chelatase (MgCh, EC 6.6.1.1) consists of the three subunits, CHLD, CHLH, and CHLI, and inserts Mg^2+^ into Proto IX to form Mg-Proto IX, while Fe-chelatase inserts Fe^2+^ into Proto IX to form heme (Beale and Weinstein, 1990; Tanaka and Tanaka, 2007). The expression of *HEMA1* encoding glutamyl-tRNA reductase 1 and *CHLH* participating in porphyrin biosynthesis is Fe responsive (Rodríguez-Celma et al., 2013; Kroh and Pilon, 2020).

In plants, plastid retrograde signals may be derived from the porphyrin pathway (Vigani et al., 2013), with different forms of porphyrins functioning as signaling molecules. The *genomes uncoupled* (*gun*) mutants *gun2*, *gun4*, and *gun5* are defective in distinct steps of porphyrin biosynthesis (Mochizuki et al., 2001; Larkin et al., 2003), supporting the idea that stress-induced accumulation of Mg-Proto IX can regulate photosynthetic genes (Strand et al., 2003; Ankele et al., 2007). CHLH (called GUN5) is a key component in both chlorophyll biosynthesis, filling the role of MgCh, and plastid-to-nucleus retrograde signaling (Shen et al., 2006). Manipulation of stomatal aperture *via* overexpression of CHLH in guard cells improves drought tolerance in Arabidopsis (Shen et al., 2006; Tsuzuki et al., 2013). CHLH was also reported to behave as an abscisic acid (ABA) receptor (Shen et al., 2006; Du et al., 2012), but there is a controversy (Müller and Hansson, 2009; Tsuzuki et al., 2011). To cope with Fe limitation, a role for ABA has been suggested in the reutilization and transport of Fe from roots to shoots in Arabidopsis (Lei et al., 2014).

In the present study, the *Arabidopsis thaliana CHLH* (*AtCHLH*) gene was chosen to prevent the cosuppression of homologous *CHLH* gene and introduced into the genome of rice to examine the consequences of increased activity of MgCh, which is a key enzyme in the flow of porphyrin metabolites, in particular, whether overexpression of *AtCHLH* enhances stress tolerance of transgenic rice plants. Many recent studies have mainly focused on how Fe uptake mechanisms respond to Fe deficiency and have identified key factors regulating root Fe uptake and translocation (Narayanan et al., 2007; Inoue et al., 2009; Kobayashi and Nishizawa, 2012). One of the plastid signals induced by Fe deficiency may be linked to porphyrin biosynthesis (Rodríguez-Celma et al., 2013; Larkin, 2016), but the exact nature of this link has not been elucidated yet. The involvement of porphyrin biosynthesis led us to investigate its possible role in determining Fe deficiency stress responses in plants. To assess the tolerance of transgenic rice heterologously expressing *AtCHLH* to Fe deficiency, we characterized the phenotypes of wild-type (WT) and transgenic plants under Fe ***-*** deficient conditions. We examined how porphyrin biosynthesis is regulated to overcome Fe deficiency and how porphyrin biosynthesis influences Fe homeostasis. We also examined if ABA is involved in regulating Fe deficiency response. Here, we report that manipulation of porphyrin biosynthesis through overexpression of *AtCHLH* alleviates Fe deficiency-induced stress in transgenic rice.

## Materials and methods

### Vector construction and rice transformation

To generate transgenic rice lines expressing *AtCHLH*, we amplified the complete coding sequence of Arabidopsis *CHLH* (At5g13630; U21294 from the Arabidopsis Biological Resource Center) using the primers 5′-ACCAACTAGTAAAATGTCGACCGCCGCTCCCA-3′ (*Spe*I site underlined) and 5′-TTTTGGTACCTTATCGATCGATCCCTTCGATCTTGTC-3′ (*Kpn*I site underlined). The resulting PCR product was digested with *Spe*I and *Kpn*I, gel-purified, and ligated into pBluescript-SK (Stratagene, Cedar Creek, TX, USA) linearized with the same restriction sites. After verifying the DNA sequence by sequencing, the *Spe*I/*Kpn*I fragment of *AtCHLH* was ligated into the same restriction sites of the pGA1611 vector (modified by the insertion of a *Spe*I restriction site) between the maize *Ubiquitin* promoter and the *Nos* 3′ terminator sequences. The resulting pGA1611:*AtCHLH* clone was transformed into *Agrobacterium tumefaciens* strain LBA4404. For rice transformation, *Agrobacterium* harboring the pGA1611:*AtCHLH* construct was co-cultured with scutellum-derived rice (*Oryza sativa* cv. Dongjin) calli as previously described (Lee et al., 2000). The independent transgenic rice lines expressing *AtCHLH* were selected based on hygromycin resistance.

### Plant growth conditions and treatments for Fe deficiency and ABA

To analyze transgene expression and porphyrin metabolism, rice seedlings of WT and homozygous transgenic lines (generations T_2_ to T_5_) were grown on soil for 4 weeks in a greenhouse at 28–30°C. For testing plant responses to nutrient deficiency, seeds of WT and two representative transgenic lines (AtCHLH-OE7 and AtCHLH-OE10) were germinated in water under dark conditions for 4 days and then hydroponically grown in different nutrient solutions under a 14-h-light/10-h-dark photoperiod with a 200 μmol m^–2^ s^–1^ photosynthetic photon flux density (PPFD) for 10 days. Growth conditions included half-strength Hoagland solution as control, water only, and water containing 50 µM Fe-EDTA, 0.5 mM MgSO_4_, or 1.25 mM NH_4_NO_3_. In other experiments to test tolerance to Fe deficiency, 1-cm emerged seedlings (to achieve uniform emergence) of WT and transgenic lines were transferred into half-strength Hoagland solution without (–Fe) or with 50 µM Fe-EDTA (+Fe) under a 14-h-light/10-h-dark photoperiod with a 200 μmol m^–2^ s^–1^ PPFD for 7 days. Samples were collected for physiological analysis and measurement of growth parameters 7 days after exposure to Fe-deficient conditions. The lengths of shoots were measured from 30–40 hydroponically grown plants per treatment using a Vernier caliper. To measure dry biomass, the shoot and root parts of plants were dried at 80°C for 48 h and then weighed.

For ABA treatment, 1-cm emerged seedlings of WT and transgenic lines were transferred to half-strength Hoagland solution under a 14-h-light/10-h-dark photoperiod with 200 μmol m^–2^ s^–1^ PPFD for 3 days. Fe deficiency was applied by transferring the seedlings to half-strength Hoagland solution without (–Fe) or with 50 µM Fe-EDTA (+Fe) for 3 days. Then, Fe-sufficient WT and transgenic seedlings were transferred to solutions without or with 0.5 µM ABA (Sigma-Aldrich, St. Louis, MO, USA) for 7 days: +Fe or +Fe+ABA. Fe-deficient WT and transgenic seedlings were also transferred to solutions without or with 0.5 µM ABA for 7 days: –Fe or –Fe+ABA.

### RNA extraction and RT-qPCR

Total RNA was prepared from leaf and root tissues using TRIzol Reagent (Invitrogen, Carlsbad, CA, USA) in accordance with the manufacturer’s instructions, and 5 µg of RNA from each sample was used for the reverse transcription reaction (*ImProm-II™* Reverse Transcription System, Promega, WI, USA). Subsequently, cDNA was used for qPCR analysis, which was carried out with a StepOnePlus™ Real-Time PCR system (Applied Biosystems, Waltham, MA, USA) using Power SYBR™ Green PCR Master Mix (Applied Biosystems) and gene-specific primers (**Supplementary Table 1**). The RT-qPCR program consisted of 2 min at 50°C, 10 min at 95°C, and 40 cycles of 15 s at 95°C and 1 min at 60°C. All reactions were set up in triplicates. Actin was used as an internal control. The WT control sample was used as a calibrator, with the expression level of the sample set to 1.

### Protein extraction, SDS-PAGE, and immunoblot analysis

Total protein was extracted with a buffer containing 12% (w/v) sucrose, 56 mM Na_2_CO_3_, 2% (*w* **/** *v*) SDS, 2 mM EDTA (pH 8.0), and 56 mM DTT. The extract was centrifuged at 12,000 × *g* and 4°C for 20 min, and the resulting supernatants were collected to obtain total soluble proteins. Soluble proteins were separated on a 12% SDS-PAGE for *At*CHLH and electroblotted onto PVDF membranes. Immunodetection was performed according to standard procedures (Roche, Basel, Switzerland). The polyclonal antibodies against *At*CHLH and *α* ***-*** tubulin were produced, by minimizing risk of cross-reactivity with *Os*CHLH (Agrisera antibody production service; **Supplementary Figure 5**), and purchased, respectively from Agrisera (Agrisera, Vännäs, Sweden).

### Determination of porphyrin contents

To measurement porphyrin contents, leaf tissue was ground in a methanol:acetone:0.1 N NaOH mixture (9:10:1, v/v/v), and the homogenate was centrifuged at 10,000 × *g* and 4°C for 10 min to remove cell debris (Lermontova and Grimm, 2006). Porphyrin was separated by high-performance liquid chromatography (HPLC) using a Novapak C_18_ column (4-µm particle size, 4.6 × 250 mm, Waters, Milford, MA, USA). Porphyrins were eluted with a gradient solvent system from 0.1 M ammonium phosphate (pH 5.8) and methanol (20:80, v/v) to 100% methanol at a flow rate of 1 mL min^–1^. The eluates were detected by a fluorescence detector (2474, Waters) at excitation and emission wavelengths of 400 nm and 630 nm, respectively, for Proto IX and 415 and 595 nm for Mg-Proto IX and Mg-Proto IX ME. For heme determination, heme was extracted and quantified as previously described (Schneegurt and Beale, 1986). Protoheme was separated by HPLC on a Novapak C_18_ column (Waters) with a solvent system of ethanol:acetic acid:water (66.5:17:16.5, v/v) and detected by a detector (SPD-M20A, Shimadzu) at 402 nm. Chlorophyll contents were determined spectrophotometrically according to the method of Lichtenthaler (1987).

### ALA-synthesizing capacity

For measurement of ALA-synthesizing capacity, leaf squares were incubated in 20 mM phosphate buffer containing 40 mM levulinic acid in the light (Papenbrock et al., 1999). Samples were homogenized, resuspended in 1 mL of 20 mM potassium phosphate buffer, pH 6.9, and centrifuged at 10,000 × *g* and 4°C for 5 min. The 500-μL supernatant was mixed with 100 μL ethylacetoacetate, boiled for 10 min, and cooled for 5 min. An equal volume of modified Ehrlich’s reagent (Mauzerall and Granick, 1956) was added, and the absorption was measured by a spectrophotometer (UV-2550, Shimadzu, Kyoto, Japan) at 553 nm.

### Assays for Mg-chelatase activity

Mg-chelatase (MgCh) was assayed as described in Lee et al. (1992) , with modifications. Leaf tissue was homogenized in homogenization buffer (50 mM Tricine (pH 7.8), 0.5 M sorbitol, 1 mM MgCl_2_, 0.1% (w/v) bovine serum albumin (BSA), and 1 mM dithiothreitol (DTT)), and centrifuged at 5,000 × *g* and 4°C for 10 min. The resulting chloroplast extracts were incubated in homogenization buffer (–BSA) containing 4 mM MgATP in a regenerating system (60 mM phosphocreatine/creatine phosphokinase, 10 units mL^−1^) and 10 m MgCl_2_. Enzyme reactions were started by adding Proto IX (in DMSO) to a final concentration of 100 µM and stopped after 1 h at 30°C. Mg-Proto IX in hexane-washed water-acetone extracts was measured by a fluorescence detector (2474, Waters) at excitation and emission wavelengths of 415 and 595 nm.

### Measurement of chlorophyll *a* fluorescence

Chlorophyll *a* fluorescence was measured *in vivo* using a pulse amplitude modulation fluorometer (JUNIOR-PAM, Walz, Effeltrich, Germany) after dark adaptation for 20 min. The minimum fluorescence (*F*_o_) at open PSII centers was determined by measuring light, while the maximum fluorescence (*F*_m_) at closed PSII centers was examined after an application of a 0.8 s pulse of saturating light (3500 μmol m^−2^ s^−1^). The *F*_v_/*F*_m_ ratio, which is the ratio of variable fluorescence (*F*_v_) to *F*_m_ after dark-adaptation and represents the maximum quantum yield of PSII, was used to assess the impairment of photosynthetic activity. Electron transport rate (ETR) was derived from photochemical quantum yield of PSII. Non-photochemical quenching (NPQ) was also quantified, as previously done by Bilger and Björkman (1990) according to the Stern–Volmer equation, NPQ = (*F*_m_ – *F*_m_΄)/*F*_m_΄.

### Perls staining of Fe

For Perls staining, seeds from WT and transgenic plants grown under the same conditions were soaked in water overnight. Dehusked seeds were then soaked in solution containing 2% (w/v) HCl and 2% (w/v) potassium hexacyanoferrate II trihydrate for 1.5 h (Ishimaru et al., 2010). The stained samples were washed with water and photographed.

### Determination of Fe content

For Fe content analysis, seeds, shoots, or roots were digested completely with 1 mL of 13 M HNO_3_ and 1 mL of 8.8 M H_2_O_2_ in an UltraWAVE instrument (Milestone Srl., Sorisole, Italy) according to the method of Ishimaru et al. (2010). Digests were analyzed for Fe content by inductively coupled plasma optical emission spectroscopy (ICP-OES) using an Optima 7300 DV instrument (Perkin Elmer, Waltham, MA, USA).

### Statistical analysis

All data are shown as means ± standard error (SE). Statistically significant differences were determined by the Duncan’s multiple range test at *P* < 0.05. The analyses were performed using SPSS software (SPSS Inc., Chicago, IL, USA).

## Results

### Heterologous expression of *AtCHLH* alters porphyrin metabolism in transgenic rice

**Figure 1:**
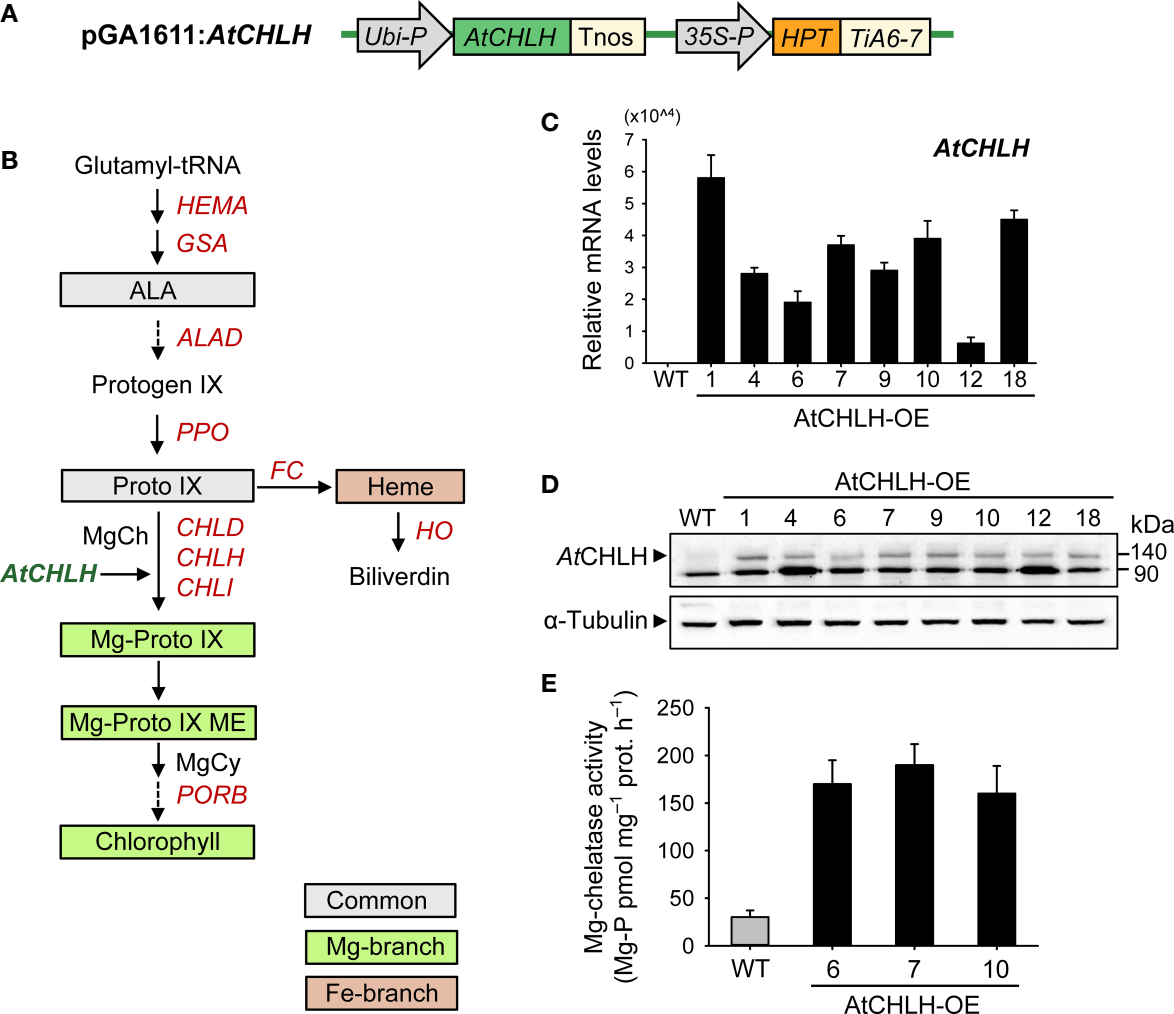
Characterization of transgenic rice plants heterologously expressing *AtCHLH*. **(A)** Schematic diagram of the T-DNA fragment in the pGA1611 binary vector. *Ubi-P*, maize *Ubiquitin* promoter; *AtCHLH*, *Arabidopsis thaliana Mg-chelatase H subunit* gene; *Tnos*, *nopaline synthase* terminator; 35S, cauliflower mosaic virus 35S promoter; *HPT*, *hygromycin phosphotransferase*; *TiA6-7*, terminator of *TiA6-7* which is a T-DNA gene in *Agrobacterium tumefaciens*. **(B)** The porphyrin biosynthetic pathway in plants showing intermediates and genes analyzed in this study. MgCy, Mg-Proto IX ME cyclase. **(C)** Expression of *AtCHLH* in leaves of transgenic lines, as determined by RT-qPCR analysis. *Actin* was used as an internal control. **(D)** Immunoblot analysis of *At*CHLH in leaves of transgenic lines, using an antibody against *At*CHLH. *Alpha****-*** tubulin was used as a loading control. **(E)** Mg-chelatase activity in WT plants and transgenic lines expressing *AtCHLH*. Mg-P, Mg-Proto IX; prot, protein. Values are means ± SE, and data from three independent experiments are presented. WT and transgenic rice plants were grown in the greenhouse under optimal conditions for 4 weeks. WT, nontransgenic wild-type rice; AtCHLH-OE1–18, transgenic rice lines overexpressing *AtCHLH*.

To increase MgCh activity in rice, we generated transgenic lines expressing *AtCHLH* under the control of the *Ubiquitin* promoter *via Agrobacterium*-mediated transformation (**Figures 1A, B**). We examined the expression of the transgene by PCR (**Supplementary Figure 1A**) and RT-qPCR in independent homozygous lines (AtCHLH-OE1–18). Strong *AtCHLH* expression was detected in all transgenic lines examined but not in nontransgenic WT plants (**Figure 1C**). We also examined the accumulation of a band with a predicted molecular weight of 140 kDa in transgenic plants by immunoblot analysis with an antibody specific to *At*CHLH protein, which localizes to chloroplasts in Arabidopsis (Gibson et al., 1996; Wu et al., 2009). We observed the 140-kDa band in immunoblots of proteins extracted from leaves, stems, and roots (**Figure 1D**; **Supplementary Figure 1B**) of all transgenic lines but not the WT, confirming the successful accumulation of *At*CHLH in transgenic rice. The band appearing at 90 kDa seems nonspecific. As CHLH is the H subunit of MgCh complex, we investigated the effects of *AtCHLH* expression on MgCh activity in the leaves of 4-week-old soil-grown transgenic plants. MgCh activity was five to six times greater in the transgenic lines AtCHLH-OE6, 7, and 10 than in WT plants, indicating that expression of *AtCHLH* substantially increases MgCh activity (**Figure 1E**). The transgenic lines were indistinguishable from the nontransgenic WT in their growth phenotype when grown under optimal conditions (**Supplementary Figure 1C**).

We next assessed the effect of heterologous *AtCHLH* expression on metabolic flux within the Mg and Fe branches for chlorophyll and heme biosynthesis, respectively. Transgenic plants exhibited a higher (58–102%) synthesizing capacity for ALA, a Proto IX precursor along the common branch, than WT plants (**Table 1**). The levels of Proto IX, a common precursor for the chlorophyll and heme branches, were not significantly different in WT and transgenic plants, but Mg-Proto IX and Mg-Proto IX ME accumulated to higher levels in transgenic plants than in WT plants (**Table 1**). These results demonstrate that the expression of *AtCHLH* in transgenic plants increases not only MgCh activity and abundance of its enzymatic product, Mg-Proto IX, but also its downstream metabolite Mg-Proto IX ME. Levels of chlorophylls and heme were not significantly different between WT and transgenic plants (**Table 1**). We selected two representative transgenic lines, AtCHLH-OE7 and AtCHLH-OE10, for further physiological experiments.

**Table 1:** Levels of metabolic intermediates in the porphyrin biosynthetic pathway in leaves of WT and *AtCHLH*-expressing transgenic rice plants grown under optimal conditions.

Plants	Porphyrin intermediates						
	ALA-synthesizing capacity (nmol g^–1^ FW h^–1^)	Proto IX (nmol g^–1^ FW)	Mg-Proto IX (nmol g^–1^ FW)	Mg-ProtoIX ME (nmol g^–1^ FW)	Chlorophyll (mg g^–1^ FW)	Heme (nmol g^–1^ FW)
WT	10.1 ± 1.6b	350 ± 55a	144 ± 28b	194 ± 21b	3.23 ± 0.18a	102 ± 7a
AtCHLH-OE6	16.0 ± 2.0ab	377 ± 49a	254 ± 31a	254 ± 16ab	3.70 ± 0.25a	111 ± 8a
AtCHLH-OE7	18.7 ± 2.2a	364 ± 45a	238 ± 20a	302 ± 30a	3.62 ± 0.26a	107 ± 16a
AtCHLH-OE10	20.4 ± 2.7a	401 ± 43a	232 ± 23a	295 ± 31a	3.60 ± 0.19a	115 ± 18a

The plants were grown under the same conditions as in **Figure 1**. AtCHLH-OE6, 7, and 10, transgenic rice lines overexpressing AtCHLH; FW, fresh weight. Values are means ± SE, and data from three independent experiments are presented. Within each column, means denoted by the same letter did not differ significantly at P < 0.05 according to Duncan’s multiple range test.

### Expression of *AtCHLH* increased tolerance to Fe deficiency in transgenic rice

**Figure 2:**
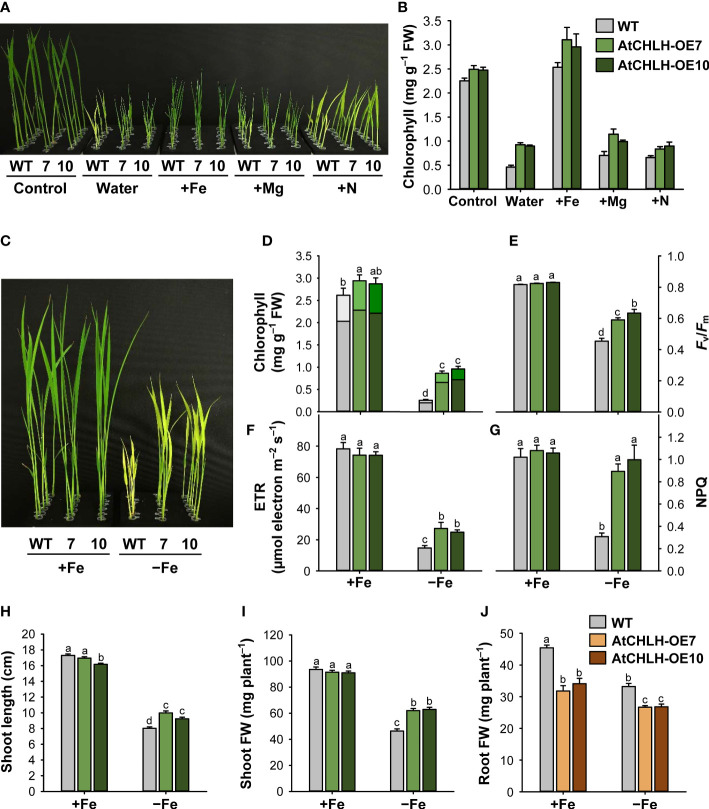
Transgenic expression of *AtCHLH* in rice enhances tolerance to Fe deficiency stress. **(A, B)** Representative phenotypes associated with typical nutrient deficiency-related symptoms **(A)** and chlorophyll contents **(B)**. WT and transgenic plants were grown hydroponically under different nutrient conditions for 10 days. Control, half-strength Hoagland solution; Water, water only; +Fe, +Mg, and +N, water supplied with 50 µM Fe-EDTA, 0.5 mM MgSO_4_, or 1.25 mM NH_4_NO_3_, respectively. **(C–J)** Rice plants heterologously expressing *AtCHLH* are more tolerant to Fe deficiency. **(C)** Fe response phenotypes in WT and *AtCHLH*-expressing plants after Fe deficiency. **(D)** Chlorophyll contents. Within each bar, lower and upper segments indicate chlorophyll *a* and *b*, respectively. **(E)** Photosynthetic performance (*F*_v_/*F*_m_). **(F)** Electron transport rate (ETR). **(G)** Non-photochemical quenching (NPQ). **(H)** Shoot length. **(I)** Shoot fresh weight. **(J)** Root fresh weight. WT and transgenic rice seedlings were transferred to half-strength Hoagland solution without or with 50 µM Fe-EDTA for 7 days. Growth characteristics of seedlings were measured and photographs were taken after 7 days of exposure to Fe deficiency. AtCHLH-OE7 and AtCHLH-OE10, transgenic lines overexpressing *AtCHLH*; +Fe, half-strength Hoagland solution with 50 µM Fe-EDTA; –Fe, half-strength Hoagland solution with no Fe-EDTA. Values are means ± SE, and data from three independent experiments are presented. Different letters indicate significant differences at *P* < 0.05 by Duncan’s multiple range test. FW, fresh weight.

Both WT and *AtCHLH* transgenic seedlings grown hydroponically in water for 10 days developed chlorosis and stunted growth of young leaves (**Figure 2A**). Interestingly, the chlorosis phenotype exhibited by the *AtCHLH* transgenic plants was more moderate than that seen in WT seedlings. To identify which missing element caused the differential chlorosis between WT and transgenic lines, we grew seedlings hydroponically in half-strength Hoagland solution (control) or in water containing a single element whose deficiency is known to cause chlorosis in seedlings: Fe, Mg, or nitrogen (N) (Uchida et al., 2000). When grown with 0.5 mM MgSO_4_ or 1.25 mM NH_4_NO_3_, both WT and transgenic seedlings still developed chlorosis and had lower chlorophyll contents, with a more severe chlorosis observed in WT seedlings than in *AtCHLH* transgenic seedlings (**Figures 2A, B**). By contrast, WT and transgenic seedlings grown in water supplemented with 50 µM Fe-EDTA resulted in no visible leaf chlorosis, although shoot growth was still impaired. *AtCHLH* transgenic seedlings had greater chlorophyll contents under these conditions than did the WT seedlings, even those grown in half-strength and Fe-replete Hoagland solution. The chlorophyll *a*/*b* ratio was lower in transgenic seedlings than that of WT seedlings under Fe-deficient conditions; 3.89, 3.23, and 3.03 in WT, AtCHLH-OE7, and AtCHLH-OE10, respectively.

Next, we grew WT and *AtCHLH* transgenic seedlings hydroponically in half-strength Hoagland solution without or with 50 µM Fe-EDTA to measure the extent of leaf chlorosis, the typical visible symptom of Fe deficiency (Terry, 1980). Although the leaves of both WT and transgenic seedlings exhibited chlorosis after transfer to Fe-free Hoagland solution for 7 days, the transgenic lines AtCHLH-OE7 and AtCHLH-OE10 showed milder chlorosis symptoms than WT seedlings (**Figure 2C**). Leaf chlorosis was accompanied by a decrease in chlorophyll contents under Fe-deficient conditions, but to a lesser extent in *AtCHLH* transgenic seedlings than in WT seedlings (**Figure 2D**). Fe deficiency also caused lower photosynthetic performance, as determined by *F*_v_/*F*_m_ values and ETR, which indicate photochemical efficiency of photosystem II (PSII) (**Figures 2E, F**). Under Fe-deficient conditions, *F*_v_/*F*_m_ values and ETR dropped more in WT seedlings than in transgenic seedlings, indicating that impairment of photosynthesis is less pronounced in transgenic seedlings. To evaluate the occurrence of protective mechanism of PSII, we measured NPQ in leaves of seedlings treated with Fe deficiency. In controls, NPQ levels were the same in WT and transgenic seedlings (**Figure 2G**). Compared to the controls, non-radiative energy dissipation through NPQ greatly decreased by 70% in WT, whereas the transgenic lines AtCHLH-OE7 and AtCHLH-OE10 did not show a noticeable decline in NPQ.

To examine the effect of *AtCHLH* expression on plant growth, we measured morphological characteristics in WT and transgenic seedlings. Under Fe-sufficient conditions, shoot length and biomass of the transgenic seedlings were comparable to those of the WT (**Figures 2H, J**). However, after 7 days of exposure to Fe deficiency, shoot length of the WT only reached about 46% that seen under Fe-sufficient conditions. Although transgenic seedlings also showed impaired shoot growth, the effect was not as pronounced, as shoot length was 24% and 15% longer in transgenic lines AtCHLH-OE7 and AtCHLH-OE10, respectively, than in WT seedlings under Fe deficiency (**Figure 2H**). Shoot biomass decreased by 50% in WT seedlings and by 32% and 31% in AtCHLH-OE7 and AtCHLH-OE10 seedlings, respectively, under Fe deficiency (**Figure 2I**). By contrast, transgenic seedlings had a lower root biomass than did WT seedlings under both Fe-sufficient and Fe-deficient conditions (**Figure 2J**). The Fe deficiency-induced decreases in total plant biomass were 42.8%, 27.8%, and 28.3% in WT, AtCHLH-OE7, and AtCHLH-OE10, respectively, resulting in a higher total biomass in transgenic seedlings (data not shown). Our results demonstrate that heterologous expression of *AtCHLH* in transgenic rice plants results in alleviation of chlorosis as well as smaller declines in chlorophyll contents, photosynthesis, and shoot growth compared to those seen in WT plants under a limited Fe pool, successfully ameliorating Fe deficiency-induced stress in rice seedlings.

### Influence of *AtCHLH* expression on metabolic control of the porphyrin biosynthetic pathway under Fe deficiency

**Figure 3:**
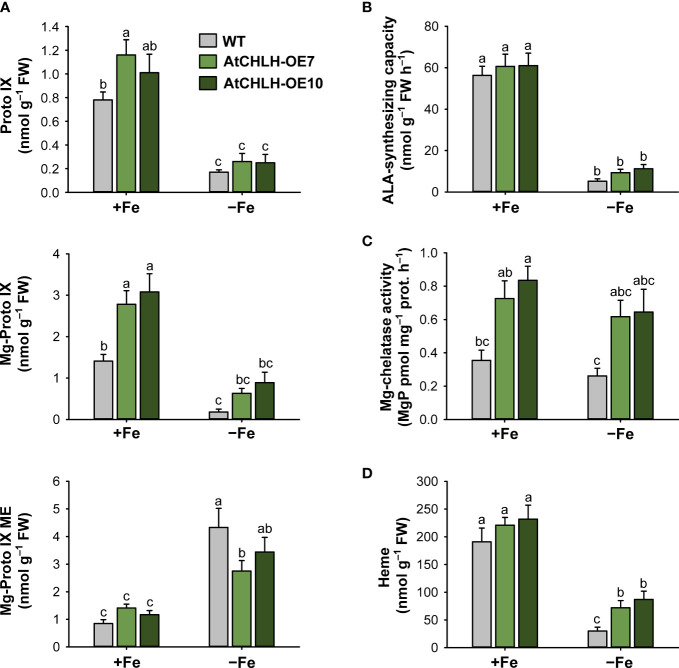
Effects of Fe deficiency on metabolic intermediates of the porphyrin biosynthetic pathway in leaves of WT and transgenic plants. **(A)** Proto IX and Mg-porphyrin intermediates. **(B)** ALA-synthesizing capacity. **(C)** Mg-chelatase activity. **(D)** Heme. WT and transgenic rice seedlings were transferred to half-strength Hoagland solution without or with 50 µM Fe-EDTA for 7 days. AtCHLH-OE7 and AtCHLH-OE10, transgenic lines overexpressing *AtCHLH*; +Fe, half-strength Hoagland solution with 50 µM Fe-EDTA; –Fe, half-strength Hoagland solution with no Fe-EDTA. Values are means ± SE, and data from three independent experiments are presented. Different letters indicate significant differences at *P* < 0.05 by Duncan’s multiple range test.

Since Fe deficiency-induced chlorosis reflects a decrease in chlorophyll levels, we examined the consequences of heterologous *AtCHLH* expression on the metabolic activities associated with porphyrin biosynthesis in seedlings grown hydroponically under Fe-sufficient and Fe-deficient conditions. In Fe-sufficient controls, *AtCHLH* transgenic seedlings accumulated more Mg-Proto IX than did WT seedlings (**Figure 3A**), in agreement with our earlier result with soil-grown plants (**Table 1**). Fe deficiency resulted in decreases in Proto IX and Mg-Proto IX levels, in addition to lower ALA-synthesizing capacity, in both WT and transgenic seedlings, with a stronger decrease in Mg-Proto IX levels in WT seedlings (**Figure 3A, B**; **Supplementary Figures 2**, **3**). By contrast, Mg-Proto IX ME accumulated to a lesser extent in Fe-deficient transgenic seedlings compared to WT seedlings. Although Fe deficiency was accompanied by lower MgCh activity in both WT and transgenic seedlings, MgCh activity remained high in transgenic seedlings relative to WT seedlings (**Figure 3C**). In the Fe-porphyrin branch, Fe deficiency lowered the heme contents in WT and transgenic seedlings, with the level of heme being greater in transgenic seedlings (**Figure 3D**; **Supplementary Figure 4**). The differences in porphyrin metabolite levels between soil-grown plants (**Table 1**) and hydroponically grown plants (**Figure 3**) may result from different growth stages (i.e., 4-week-old plants versus young seedlings) and conditions (i.e., light level and soil versus hydroponics).

**Figure 4:**
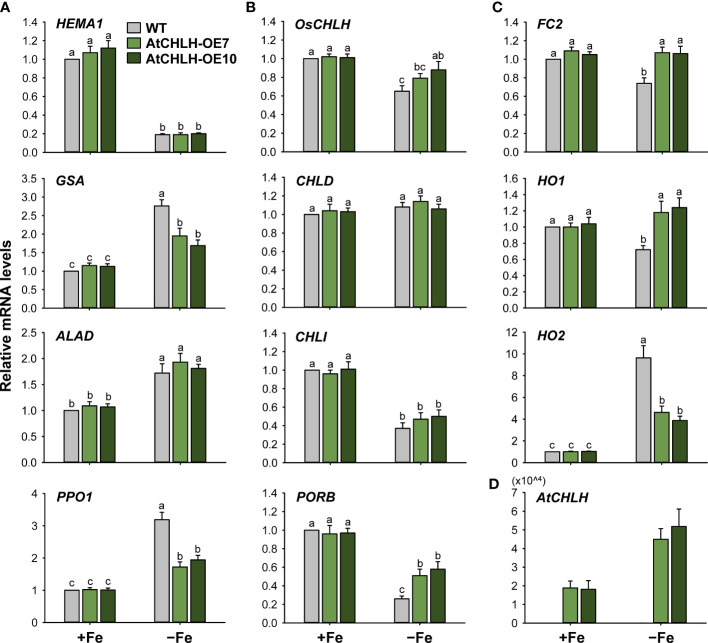
Fe deficiency-induced changes in the expression of genes encoding enzymes from the porphyrin biosynthetic pathway in leaves of WT and transgenic plants. **(A)** Common branch. **(B)** Mg-porphyrin branch. **(C)** Fe-porphyrin branch. **(D)** *AtCHLH* transgene in Mg-porphyrin branch. The plants were subjected to the same treatments as in **Figure 3**. Treatment notations are the same as in **Figure 3**. Total RNAs were extracted and reverse transcribed. The resulting first-strand cDNAs were used as templates for qPCR, using *Actin* as an internal control. All relative expression levels were normalized to the +Fe WT control values, which were set to 1. Values are means ± SE, and data from three independent experiments are presented.

To explore the molecular mechanisms underlying these changes in porphyrin metabolism induced by Fe deficiency, we determined relative transcript levels for key genes in porphyrin biosynthesis by RT-qPCR. Under Fe-sufficient conditions, we observed no significant difference in expression for most porphyrin biosynthetic genes between WT and transgenic seedlings (**Figure 4**). In the common pathway, we analyzed two genes encoding enzymes critical for ALA-synthesizing activity, *HEMA1* and *glutamate 1-semialdehyde aminotransferase* (*GSA*) (Beale and Weinstein, 1990). Relative *HEMA1* expression decreased by approximately 80% upon Fe deficiency in WT and transgenic seedlings compared to Fe-sufficient conditions (**Figure 4A**). By contrast, relative transcript levels of *GSA*, *ALAD* (encoding ALA dehydratase), and *PPO1* increased by 50–219% under Fe-deficient conditions in WT and transgenic seedlings relative to Fe-sufficient conditions, with a greater increase for *GSA* and *PPO1* in WT seedlings.

We next assessed genes belonging to the Mg-porphyrin or Fe-porphyrin branch as a possible explanation for the observed changes in Mg-porphyrins and heme under Fe-deficient conditions. *OsCHLH* and *CHLI*, which encode the H and I subunits of MgCh, respectively, were downregulated in WT and transgenic seedlings experiencing Fe-deficient conditions, with a smaller reduction in *OsCHLH* transcript levels in transgenic seedlings; *CHLD* remained constant in all lines and in both growth conditions (**Figure 4B**). Fe deficiency downregulated *protochlorophyllide oxidoreductase B* (*PORB*), with transcript levels in WT seedlings reaching about 26% of those seen in Fe-sufficient conditions; relative *PORB* transcript levels dropped only 40–47% in Fe-deficient transgenic seedlings compared to Fe-sufficient seedlings. In the Fe-porphyrin branch, *Fe-chelatase 2* (*FC2*), encoding the plastidic isoform of Fe-chelatase (Beale and Weinstein, 1990), was downregulated by about 26% in response to Fe deficiency in WT seedlings but remained constant in transgenic seedlings regardless of growth condition (**Figure 4C**). Under Fe-deficient conditions, *HO1* encoding heme oxygenase, which catalyzes the formation of biliverdin-IXα, carbon monoxide (CO), and Fe^2+^ through the oxidation of heme (Tanaka and Tanaka, 2007), remained constant in transgenic seedlings but downregulated by 28% in WT seedlings. *HO2* exhibited a 10-fold increase in transcript levels in Fe-deficient WT seedlings but only reached 4-fold higher levels in transgenic seedlings under the same conditions. In transgenic seedlings, *AtCHLH* transcript levels increased up to 3-fold upon Fe deficiency (**Figure 4D**), showing that *AtCHLH* is a Fe deficiency-responsive gene. Overall, porphyrin metabolism was differentially modulated by Fe deficiency stress between WT and transgenic seedlings. These results indicate that heterologous expression of *AtCHLH* reprograms porphyrin metabolism in transgenic seedlings under Fe-deficient conditions.

### *AtCHLH* expression-induced alterations in regulation of Fe homeostasis-related genes under Fe deficiency

**Figure 5:**
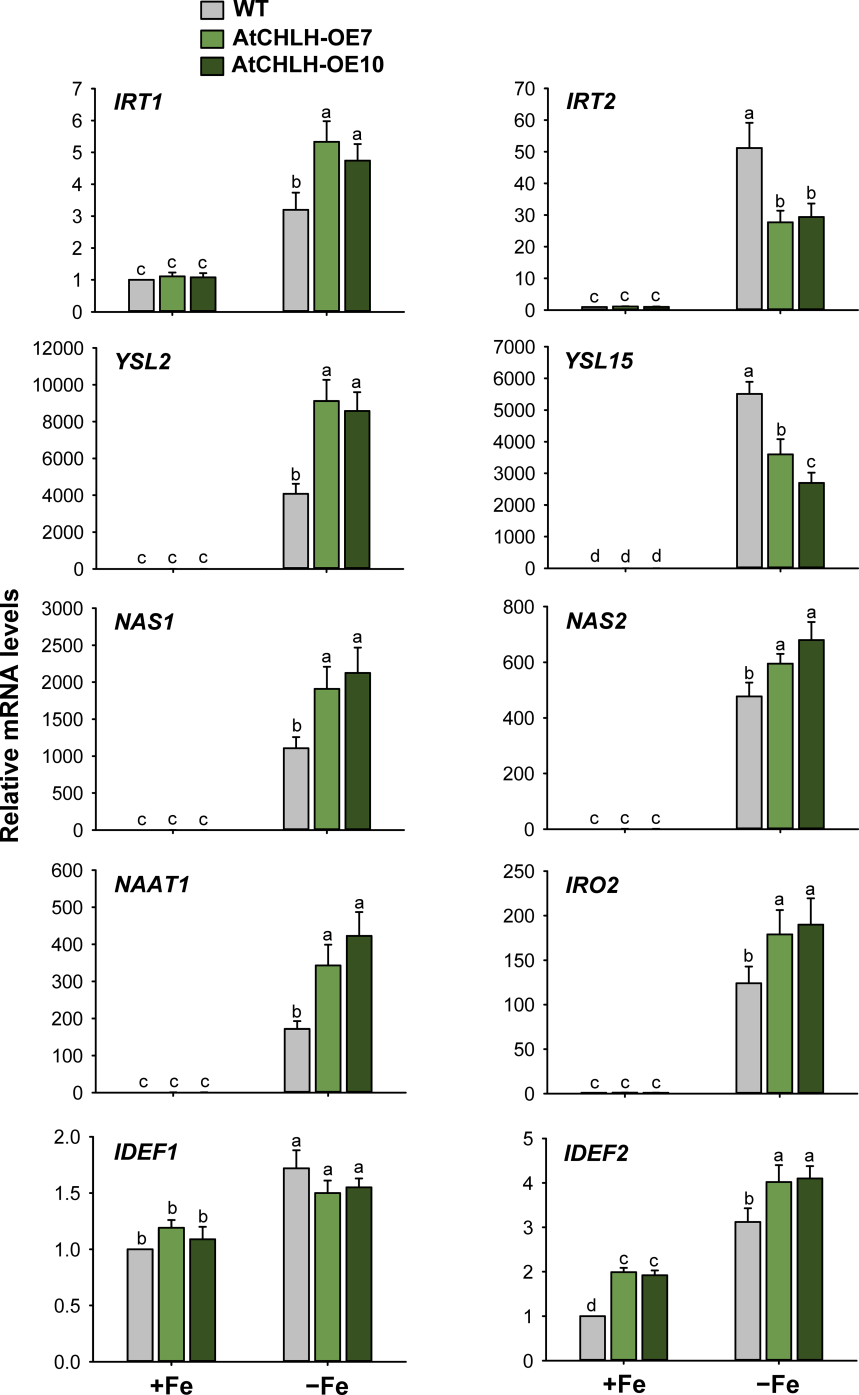
Expression of Fe transporter and Fe deficiency-induced transcription factor genes in leaves of WT and transgenic plants. The plants were subjected to the same treatments as in **Figure 3**. Treatment notations are the same as in **Figure 3**. The general steps performed during the RT-qPCR experiment, from RNA isolation to data analysis, are outlined in **Figure 4**. Values are means ± SE, and data from three independent experiments are presented. Different letters indicate significant differences at *P* < 0.05 by Duncan’s multiple range test.

**Figure 6:**
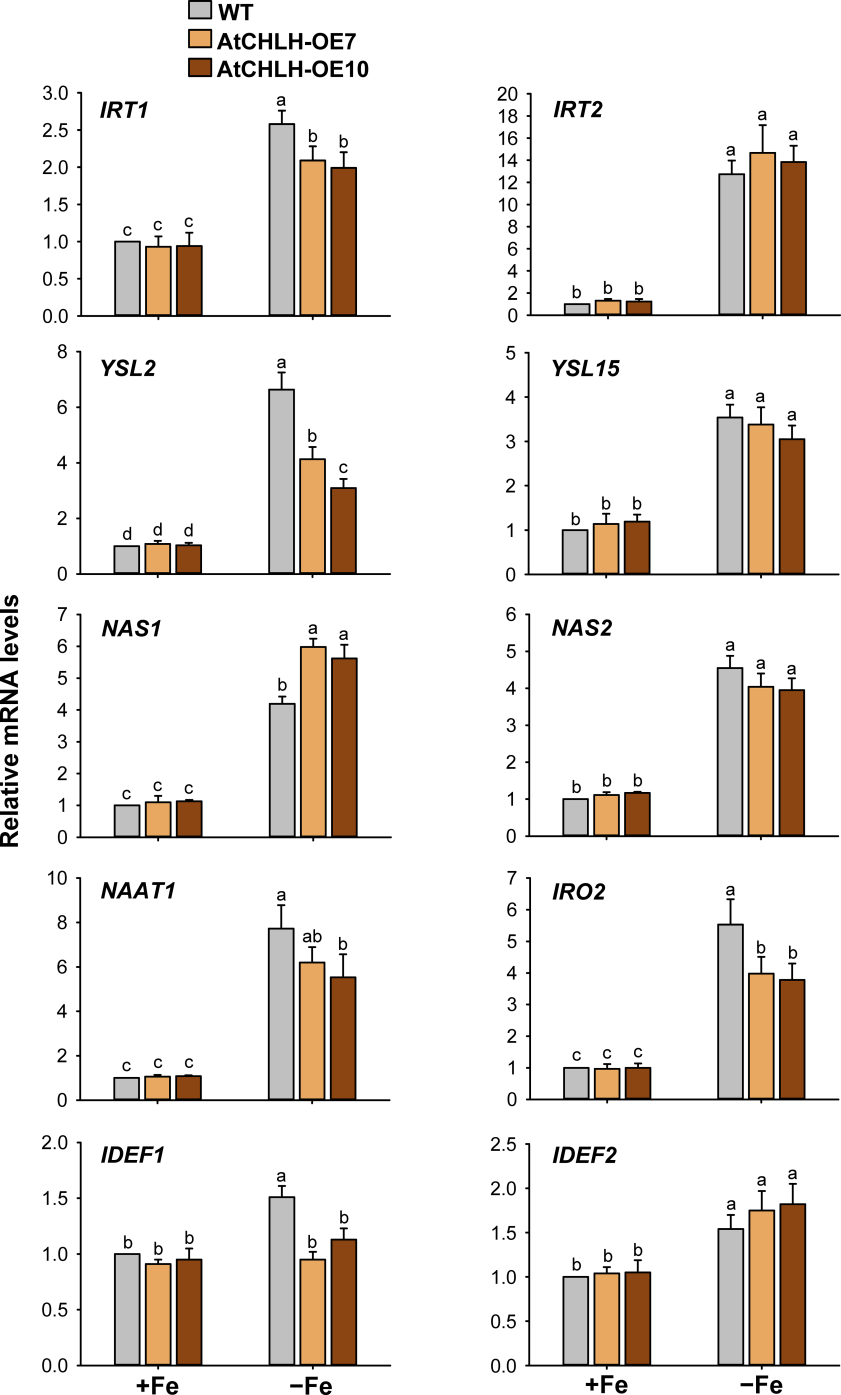
Expression of Fe transporter and Fe deficiency-induced transcription factor genes in roots of WT and transgenic plants. The plants were subjected to the same treatments as in **Figure 3**. Treatment notations are the same as in **Figure 3**. The general steps performed during the RT-qPCR experiment, from RNA isolation to data analysis, are outlined in **Figure 4**. Values are means ± SE, and data from three independent experiments are presented. Different letters indicate significant differences at *P* < 0.05 by Duncan’s multiple range test.

When subjected to Fe deficiency, plants need to manipulate Fe uptake and translocation efficiently. To determine whether *AtCHLH* influences Fe uptake and/or homeostasis in transgenic rice, we compared the expression of representative Fe transporter genes between WT and transgenic seedlings under Fe-deficient conditions. In Fe-deficient leaves, the expression of Fe transporter gene *IRT1* showed a 3-fold increase in WT seedlings and about 5-fold increase in transgenic seedlings, whereas *IRT2* exhibited the opposite pattern, with a higher rise upon Fe deficiency in WT relative to transgenic seedlings (**Figure 5**). Transcript levels of the other Fe transporter genes, *YSL2* and *YSL15*, markedly increased in WT and transgenic leaves under Fe-deficient conditions; a greater increase of *YSL2* and *YSL15* in transgenic and WT leaves, respectively. The genes that encode key enzymes for MA biosynthesis including *NAS1*, *NAS2*, and *NAAT1* greatly upregulated in Fe-deficient leaves of WT and transgenic seedlings, with greater upregulation in transgenic leaves. Transcript levels of *IRO2* encoding the iron-deficiency-inducible bHLH transcription factor 2 greatly increased in WT and transgenic leaves in response to Fe deficiency, with a greater increase in transgenic leaves. The genes *IDEF1* and *IDEF2* belong to ABI3 transcription factor family and NAC-family transcription factor, respectively (Kobayashi et al., 2007; Ogo et al., 2008), showed smaller increases in leaves of WT and transgenic seedlings, compared to those of other Fe-related genes (**Figure 5**). In Fe-deficient roots, *IRT1* and *IRT2* were strongly upregulated in WT and transgenic seedlings, with a slightly greater induction for *IRT1* observed in WT seedlings (**Figure 6**). Under Fe-deficient conditions, increased levels of *NAAT1*, *YSL2*, and *IRO2* were greater in WT roots than in transgenic roots, whereas increased level of *NAS1* was greater in transgenic roots. Induction of *IRT2*, *NAS2*, and *YSL15* upon Fe deficiency was similar between WT and transgenic roots. In response to Fe deficiency, transcript level of *IDEF1* increased in WT roots but remained constant in transgenic roots, while *IDEF2* slightly increased in WT and transgenic roots (**Figure 6**).

**Figure 7:**
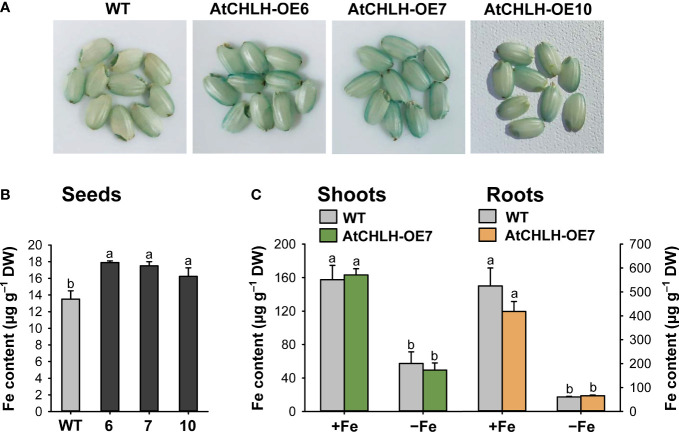
Fe contents in different tissues of WT and transgenic plants. **(A)** Fe localization in rice seeds by Perls staining. **(B)** Fe contents in seeds by ICP-OES. **(C)** Fe contents in shoots and roots under Fe-sufficient and Fe-deficient conditions by ICP-OES. For obtaining shoots and roots, the plants were subjected to the same treatments as in **Figure 3**. Treatment notations are the same as in **Figure 3**. Values are means ± SE, and data from three independent experiments are presented. Different letters indicate significant differences at *P* < 0.05 by Duncan’s multiple range test. DW, dry weight.

As Fe is taken up by the roots, we explored whether altered expression of Fe homeostasis-related genes in transgenic seedlings influenced Fe translocation from roots to shoots and seeds. We employed the Fe stain Perls Prussian blue on seeds, which confirmed that Fe contents were higher in transgenic seeds compared to WT seeds, as indicated by the stronger blue color on the surface of transgenic seeds (**Figure 7A**). We also conducted ICP-OES to measure the contents of Fe in seeds. Fe contents were higher by 9–33% in transgenic seeds compared to WT seeds (**Figure 7B**). The contents of Fe were obtained in seedlings grown hydroponically under Fe-sufficient or Fe-deficient conditions. Shoots and roots of WT and transgenic seedlings accumulated comparable levels of Fe when grown under Fe-sufficient conditions (**Figure 7C**). In response to Fe deficiency, Fe levels similarly dropped in shoots and roots of both WT and transgenic seedlings.

### Effects of ABA on Fe deficiency-induced responses and regulation of CHLH

**Figure 8:**
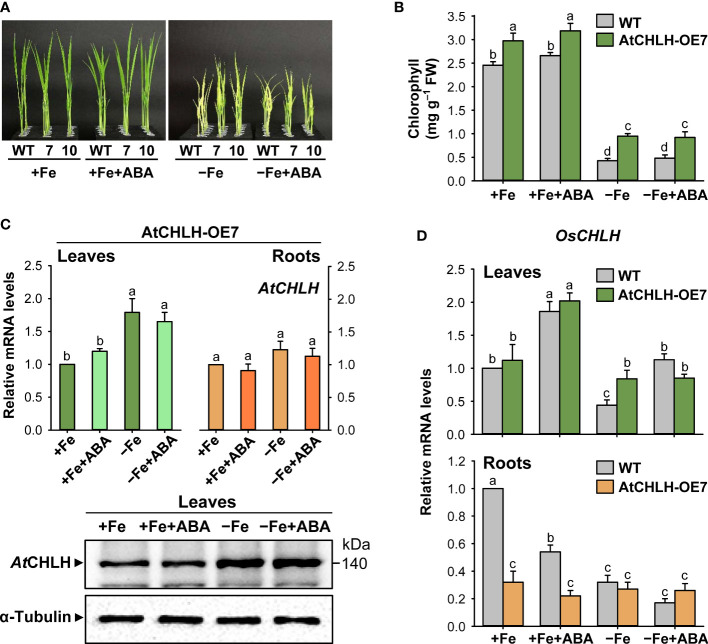
Effect of exogenous ABA on Fe deficiency responses and regulation of CHLH. **(A)** Representative phenotypes of Fe-sufficient and Fe-deficient WT and transgenic plants without or with ABA treatment. **(B)** Chlorophyll contents. **(C)** *AtCHLH* transcript levels and *At*CHLH protein abundance in transgenic plants. *Alpha***-**tubulin was used as a loading control in immunoblot analysis. **(D)** *OsCHLH* transcript levels in leaves and roots of WT and transgenic plants. The general steps performed during the RT-qPCR experiment, from RNA isolation to data analysis, are outlined in **Figure 4**. WT and transgenic plants were grown in half-strength Hoagland solution with 50 µM Fe-EDTA (+Fe) or no Fe-EDTA (–Fe) for 3 days. Then, Fe-sufficient WT and transgenic seedlings were transferred to solutions without or with 0.5 µM ABA for 7 days: +Fe or +Fe+ABA. Fe-deficient WT and transgenic seedlings were also transferred to solutions without or with 0.5 µM ABA for 7 days: –Fe or –Fe+ABA. Values are means ± SE, and data from three independent experiments are presented. Different letters indicate significant differences at *P* < 0.05 by Duncan’s multiple range test.

To explore a possible role for ABA in the Fe deficiency responses, Fe-sufficient and Fe-deficient rice seedlings were transferred to different solutions without or with 0.5 µM ABA for 7 days. This exogenous ABA treatment did not induce noticeable changes in leaf phenotypes or chlorophyll contents in either WT or transgenic seedlings under any growth condition (**Figures 8A, B**). We also compared the effects of ABA treatment on expression of *AtCHLH* and *OsCHLH* under Fe-sufficient and Fe-deficient conditions. *At*CHLH transcript level and protein abundance in transgenic leaves did not significantly change following ABA treatment under Fe-sufficient or Fe-deficient conditions (**Figures 8C**). In transgenic roots, ABA did not induce any changes in *AtCHLH* transcript levels in both conditions (**Figure 8C**). By contrast, exogenous ABA treatment upregulated endogenous *OsCHLH* transcript levels in leaves of WT and transgenic seedlings about 2-fold under Fe-sufficient conditions (**Figure 8D**). While WT leaves experienced a 56% drop in *OsCHLH* transcript levels upon Fe deficiency, exogenous application of ABA to Fe-deficient seedlings returned *OsCHLH* transcripts to levels comparable to those of Fe-sufficient WT leaves. However, ABA treatment had no effect on *OsCHLH* expression in Fe-deficient transgenic leaves. Similarly, both Fe deficiency and exogenous ABA additively repressed *OsCHLH* expression in WT roots, but not in transgenic roots, in which *OsCHLH* expression remained constant.

## Discussion

Fe deficiency induces chlorosis in young leaves, which causes severe reductions in yield and grain quality at the adult stage (Guo et al., 2017; Kobayashi et al., 2019). Here, we present a new approach to ameliorate Fe deficiency-induced stress of plants through manipulation of porphyrin biosynthesis. Transgenic rice plants expressing an introduced *AtCHLH* substantially increased MgCh activity by up to 6-fold and abundance of its product, Mg-Proto IX, by 60–75% compared to WT plants (**Figure 1E**; **Table 1**), showing that transgenic *At*CHLH is functional and reprograms porphyrin metabolism in transgenic rice. When grown hydroponically without any nutrients, transgenic seedlings notably alleviated chlorosis compared to WT seedlings (**Figure 2A**). Exogenous Fe supply fully reversed the nutrient deficiency-induced chlorosis in WT and transgenic seedlings. This points to Fe deficiency as the main cause of the observed chlorosis.

After exposure to Fe deficiency stress, *AtCHLH*-expressing transgenic seedlings showed mitigation of chlorosis and a lesser decrease in chlorophyll contents relative to WT seedlings (**Figures 2C, D**), reflecting the enhanced survival of the transgenic seedlings under Fe deficiency. Even in Fe-sufficient controls, transgenic seedlings accumulated slightly more chlorophyll than WT seedlings (**Figure 2D**), which may be due to increased metabolic flux to the chlorophyll branch. Photosynthesis has an extensive Fe quota, with the largest sinks for Fe being PSI and PSII in chloroplasts (Rodríguez-Celma et al., 2013). Low Fe supply results in a reduction of the number of light-harvesting antenna complexes (LHCII) at PSII caused by lower PSII pigments, as their biosynthesis requires Fe (Terry, 1980; Morales et al., 1991). In response to Fe deficiency, *F*_v_/*F*_m_ values and ETR, which are positively correlated with PSII organization, decreased in WT and transgenic seedlings, but to a lesser extent in transgenic seedlings (**Figures 2E, F**). The reduction in PSII under Fe deficiency may be due to photoinhibition caused by an imbalance in the excitation of electron transfer between PSI and PSII rather than a direct effect of Fe deficiency. Some studies on Fe depletion in plants reported that PSI was the major target of Fe deficiency (Timperio et al., 2007; Higuchi and Saito, 2022). NPQ reflects thermal dissipation of excitation energy in PSII antennae, which depends on acidification of the lumen and thus electron transport activity (Schreiber et al., 1994). Under Fe-deficient conditions, WT seedlings strongly diminished NPQ by 70% in comparison to those in the control conditions, whereas transgenic seedlings were able to maintain NPQ (**Figures 2G**), indicating a higher photoprotective capacity in transgenic seedlings during Fe deficiency. The LHCII of PSII is directly involved in NPQ and the aggregation of LHCII is proposed to be involved in quenching (Shukla et al., 2020). A possible reason for the higher NPQ in the transgenic seedlings may be high LHCII levels, as indicated by 17–22% lower chlorophyll *a*/*b* ratio in Fe-deficient transgenic seedlings than that of WT seedlings. The higher chlorophyll may be distributed more to LHCII, thereby suppressing the NPQ decrease due to the deficiency of Fe in transgenic seedlings. Sustained photosynthetic efficiency and photoprotection in Fe-deficient transgenic seedlings may help explain their longer shoots and higher shoot biomass compared to WT seedlings under the same conditions (**Figures 2H, I**). These data indicate that the alleviation of Fe deficiency phenotypes in the transgenic seedlings is associated with the expression of *AtCHLH*, although little is known about the involvement of porphyrin biosynthetic pathway in Fe deficiency responses.

Next, we examined Fe deficiency-induced metabolic changes in porphyrin biosynthesis to elucidate the role of porphyrin in Fe deficiency responses. *GSA*, *ALAD*, and *PPO1* were markedly upregulated in Fe-deficient WT and transgenic seedlings, perhaps in an effort to compensate for the loss of chlorophyll, while *HEMA1* was downregulated (**Figure 4A**). This result indicates that ALA biosynthesis undergoes transcriptional regulation in response to Fe deficiency, but this does not translate into a sustained capacity to synthesize ALA, resulting in the severe decrease in Proto IX levels in Fe-deficient seedlings (**Figure 3**). The Mg-porphyrin branch starts with the synthesis of Mg-Proto IX catalyzed by MgCh (Beale and Weinstein, 1990; Tanaka and Tanaka, 2007). During Fe deficiency, the decrease in Mg-Proto IX levels was partially caused by the downregulation of *OsCHLH* and *CHLI* in WT and transgenic seedlings (**Figures 3A**, **4B**). The downregulation of *OsCHLH* and *HEMA1* upon Fe deficiency is in accordance with the results observed in Arabidopsis (Yang et al., 2010; Rodríguez-Celma et al., 2013). Other environmental stresses including drought and chilling also influenced metabolites from the porphyrin biosynthetic pathway through their scavenging to cope with excited-state dynamics of porphyrins (Phung et al., 2011; Phung and Jung, 2015). Under Fe deficiency, the heterologous expression of *AtCHLH* mitigated the decrease in Mg-Proto IX levels, which may contribute to the higher chlorophyll contents and photosynthetic capacity in transgenic seedlings than WT seedlings. Among the genes in the Fe*-*porphyrin branch, expression levels of *FC2* remained constant in transgenic seedlings regardless of growth conditions, which may partially account for their less pronounced decrease in heme contents under Fe-deficient conditions, whereas *FC2* dropped by about 25% in WT seedlings (**Figures 3**, **4C**). HO1 is suggested as a regulator for Fe availability in Fe-stress cells because heme may be the source for Fe mobilization within cells (Kong et al., 2010). The antioxidant potential of a well-known antioxidant enzyme HO1 is primarily due to its catalytic reaction byproducts, CO and biliverdin (Singh and Bhatla, 2022). The stable expression levels for *FC2* and *HO1* in transgenic seedlings (**Figure 4C**) seem to partly contribute to the alleviation of Fe deficiency-induced stress. Under Fe deficiency, the increased expression of *HO2* in WT and transgenic seedlings may imply a high demand for the antioxidative protection. In contrast to our results, enzymes in the Fe*-*porphyrin branch were not transcriptionally regulated by Fe deficiency in a previous study (Rodríguez-Celma et al., 2013).

Because major Fe sinks are in the shoots, a systemic shoot-to-root signal must exist to coordinate proper Fe supply (Vert et al., 2003; García et al., 2013). Enzymes within the porphyrin synthesis pathway have been suggested as viable candidates for chloroplast Fe sensing in plants because the porphyrin pathway is known to have a component of retrograde signaling (Larkin, 2016). The Fe deficiency response in the nucleus is thought to be regulated by plastidic Fe signals as well as other related compounds such as products of primary metabolism (Vigani et al., 2013). While *AtCHLH* transcript and *At*CHLH protein accumulated in leaves of Fe-deficient transgenic seedlings, expression levels of *OsCHLH* were comparable in WT and transgenic seedlings under both Fe-sufficient and Fe-deficient conditions, such that the higher MgCh activity measured in transgenic plants primarily stems from accumulation of Fe-responsive *At*CHLH (**Figures 3**, **4**, **8**). That Mg-Proto IX is more abundant in Fe-deficient transgenic seedlings compared to WT seedlings (**Figure 3A**) suggests its possible role in sensing Fe deficiency. Mg-Proto IX has been suggested to act as a signaling molecule in one of the signaling pathways between the chloroplast and nucleus (Mochizuki et al., 2001; Strand et al., 2003; Zhang et al., 2011). The accumulation of Mg-Proto IX ME in Fe-deficient WT and transgenic seedlings (**Figure 3**) appears to result from the loss of activity of the Fe metalloprotein Mg-Proto IX ME cyclase under Fe-deficient conditions (Spiller et al., 1982). The Mg branch of porphyrin biosynthesis is suggested to be responsible for sensing Fe status (Rodríguez-Celma et al., 2013; Kroh and Pilon, 2020). In our study, marked changes in levels of metabolites and gene expression in porphyrin biosynthetic pathway were observed in both Mg and Fe branches, suggesting that both branches may involve in sensing Fe status.

After Fe uptake from the root, Fe translocates to leaves to support chlorophyll biosynthesis and photosynthesis (Spiller et al., 1982; Pushnik et al., 1984). The Fe^2+^ transporter gene IRT1 is known to accumulate to high levels in rice roots upon Fe starvation (Bughio et al., 2002; Ishimaru et al., 2006). The high transcript levels of *IRT1* and *IRT2* were induced by Fe deficiency in both leaves and roots of WT and transgenic seedlings (**Figures 5**, **6**). Under Fe-deficient conditions, the Fe(II)- and manganese(II)-nicotianamine transporter gene *YSL2*, which is thought to be involved in the internal transport of Fe within the plant body (Koike et al., 2004), exhibited greater upregulation in transgenic leaves and WT roots (**Figures 5**, **6**). The Fe(III)-DMA transporter gene *YSL15*, whose encoded transporter is responsible for the uptake of Fe^3+^-siderophore complexes from the rhizosphere and transport of Fe *via* the phloem (Inoue et al., 2009), was strongly induced by Fe deficiency in leaves and roots of WT and transgenic seedlings, with a greater increase in WT leaves (**Figures 5**, **6**). Upregulation of *IRT* and *YSL*, which play an important role in Fe homeostasis, indicates an effort to facilitate Fe movement from roots to leaves under Fe-deficient conditions. The bHLH transcription factor IRO2 is a positive regulator of most genes known to be involved in Fe(III)-DMA uptake and translocation (Ogo et al., 2007). In response to Fe deficiency, a marked increase in transcript levels of *IRO2* was greater in transgenic leaves than WT leaves, in agreement with greater upregulation of DMA biosynthesis genes including *NAS1*, *NAS2*, and *NAAT1* in transgenic leaves (**Figure 5**). However, Fe deficiency-induced increases of *NAAT1*, *YSL2*, and *IRO2* were greater in WT roots than transgenic roots (**Figure 6**). DMA is responsible not only for Fe uptake from the rhizosphere, but also for internal Fe translocation (Kobayashi et al., 2019).

The Fe deficiency signal is thought to be initiated from the shoots to induce root Fe uptake (Vert et al., 2003; García et al., 2013). IDEF1 and hemerythrin motif-containing HRZs have recently emerged as candidate Fe sensors because of their functions as potent regulators of Fe deficiency responses and their Fe-binding properties (Kobayashi et al., 2012, Kobayashi et al., 2013; Kobayashi and Nishizawa, 2014). *IDEF1* and *IDEF2*, whose encoded transcription factors mediate the induction of Fe-related genes under Fe-deficient conditions, were constitutively expressed in rice roots and leaves (Kobayashi et al., 2007; Ogo et al., 2008; Kobayashi et al., 2010). In our study, transcript levels of *IDEF1* and *IDEF2* exhibited increases in response to Fe deficiency in most treatments, although the degree of increase was minor compared to other Fe homeostasis-related genes (**Figures 5**, **6**). This discrepancy could be due to young seedlings at an early growth stage employed for our study. Overall, markedly greater upregulation of Fe homeostasis-related genes was observed in Fe-deficient leaves than Fe-deficient roots because their relative mRNA levels were extremely low in Fe-sufficient control leaves. In addition, transgenic seeds exhibited the stronger blue color on the surface of transgenic seeds and 9–33% higher Fe contents compared to WT seeds (**Figures 7A, B**). However, shoots and roots accumulated comparable levels of Fe between WT and transgenic seedlings when grown under Fe-sufficient and Fe-deficient conditions (**Figure 7C**), indicating that higher Fe contents in transgenic seeds appear not to influence the Fe contents of shoots and roots. There is a possibility that higher Fe contents in transgenic seeds might contribute to the alleviation of Fe deficiency symptoms observed in transgenic seedlings (**Figure 2**), because seedlings are also supplied with nutrition from the cotyledons. Therefore, further studies on longer-term cultivation are necessary in order to clarify the possibility. Our results showed that the transcriptional control of Fe homeostasis-related genes plays an important role in Fe deficiency responses. Particularly, greater expression levels of *YSL2*, DMA biosynthesis-related genes (*NAS1*, *NAS2*, and *NAAT1*), *IRO2*, and *IDEF2* in transgenic leaves than WT leaves may partly contribute to alleviation of Fe deficiency stress.

While ABA participates in Fe homeostasis and alleviation of Fe deficiency (Lei et al., 2014; Guo et al., 2017; Zhang et al., 2020), its exact role in Fe homeostasis remains unknown. Since exogenous application of ABA did not rescue Fe deficiency phenotypes including the chlorosis and growth defects of WT and transgenic seedlings observed upon Fe deficiency (**Figures 8A, B**), *At*CHLH is unlikely to affect Fe deficiency responses *via* ABA signaling. Transcript levels of *IDEF1*, which belongs to the ABI3 family of transcription factors involved in mediating responses to ABA (Kobayashi et al., 2007), were similar in WT and transgenic seedlings, except for a slightly higher level of *IDEF1* in Fe-deficient WT roots (**Figures 5**, **6**). *At*CHLH function was also not correlated with ABA responses in Arabidopsis guard cells (Ibata et al., 2016). However, other studies reveal that *At*CHLH binds ABA and functions in ABA signaling through regulating seed germination and post-germination growth (Shen et al., 2006; Wu et al., 2009). This prompted us to examine whether ABA regulates expression of endogenous *OsCHLH* and transgene *AtCHLH* during Fe deficiency. In leaves and roots of transgenic seedlings, the expression levels of the *AtCHLH* did not change in response to exogenous ABA under Fe-sufficient or Fe-deficient conditions (**Figures 8C, D**), showing that *AtCHLH* is not responsive to ABA. By contrast, endogenous *OsCHLH* transcript levels rose in an ABA-dependent manner in the leaves of Fe-sufficient WT and transgenic seedlings, as well as Fe-deficient WT seedlings (**Figure 8E**). Although the role of CHLH in roots is unclear, *OsCHLH* responded to ABA only in Fe-sufficient WT roots. Our results do not confirm whether CHLH is involved in Fe deficiency response *via* ABA signaling.

Transgenic plants overexpressing *AtCHLH* alleviated Fe deficiency-induced chlorosis and maintained higher chlorophyll content, photosynthetic function, photoprotective capacity, and shoot biomass under a limited Fe pool compared to WT plants, which may be due to increased metabolic flux to the chlorophyll branch through higher MgCh activity. These results show that manipulation of porphyrin biosynthesis through expression of *AtCHLH* enhances the capacity to cope with Fe limitation, alleviating Fe deficiency-induced stress in transgenic rice. In transgenic plants, transcriptional and translational upregulation of *At*CHLH took place under Fe-deficient conditions, indicating the function of CHLH in protecting plants from Fe deficiency. Then, a sustained MgCh activity in transgenic plants led to a higher level of Mg-Proto IX than WT plants under Fe deficiency, which could be involved in metabolic reprogramming of porphyrin biosynthesis and Fe deficiency responses possibly via transcriptional regulation of Fe homeostasis-related genes. Under Fe deficiency, levels of porphyrin metabolites were greatly affected by Fe limitation, with the differential modulation of porphyrin metabolism between WT and transgenic plants, indicating the reprogramming of porphyrin biosynthesis in transgenic plants by heterologous expression of *AtCHLH*. In addition to the stable expression levels for *FC2* and *HO1*, greater upregulation of DMA biosynthesis-related genes and *IRO2* as well as *YSL2* and *IDEF2* in transgenic leaves may be partly implicated in alleviating Fe deficiency-induced stress. However, the mechanism underlying how overexpression of *AtCHLH* mediates the mitigation of Fe deficiency stress through transcriptional control of Fe homeostasis genes is still not clear. Based on our findings that transgenic rice expressing *AtCHLH* alleviates Fe deficiency-induced stress, we suppose that the regulatory mechanism for porphyrin metabolism is part of the complex protective systems against Fe deficiency stress. Our study also provides new insight into a possible crosstalk between the porphyrin biosynthetic pathway and Fe deficiency signaling.

## Data availability statement

The original contributions presented in the study are included in the article/**Supplementary Material**, further inquiries can be directed to the corresponding author.

## Author contributions

SJ conceived and designed research. LT and J-GK performed the experiments. SJ and LT analyzed the data. SJ drafted the manuscript with contribution of all the authors. All authors contributed to the article and approved the submitted version.
