# Supplementary material for: Expression of the Arabidopsis Mg-chelatase H subunit alleviates iron deficiency-induced stress in transgenic rice
Source: Front Plant Sci. 2023 Mar 2;14:1098808. doi: 10.3389/fpls.2023.1098808 (PMC10017980; doi:10.3389/fpls.2023.1098808)
Supplement: Supplementary file 1 [file DataSheet_1.pdf]

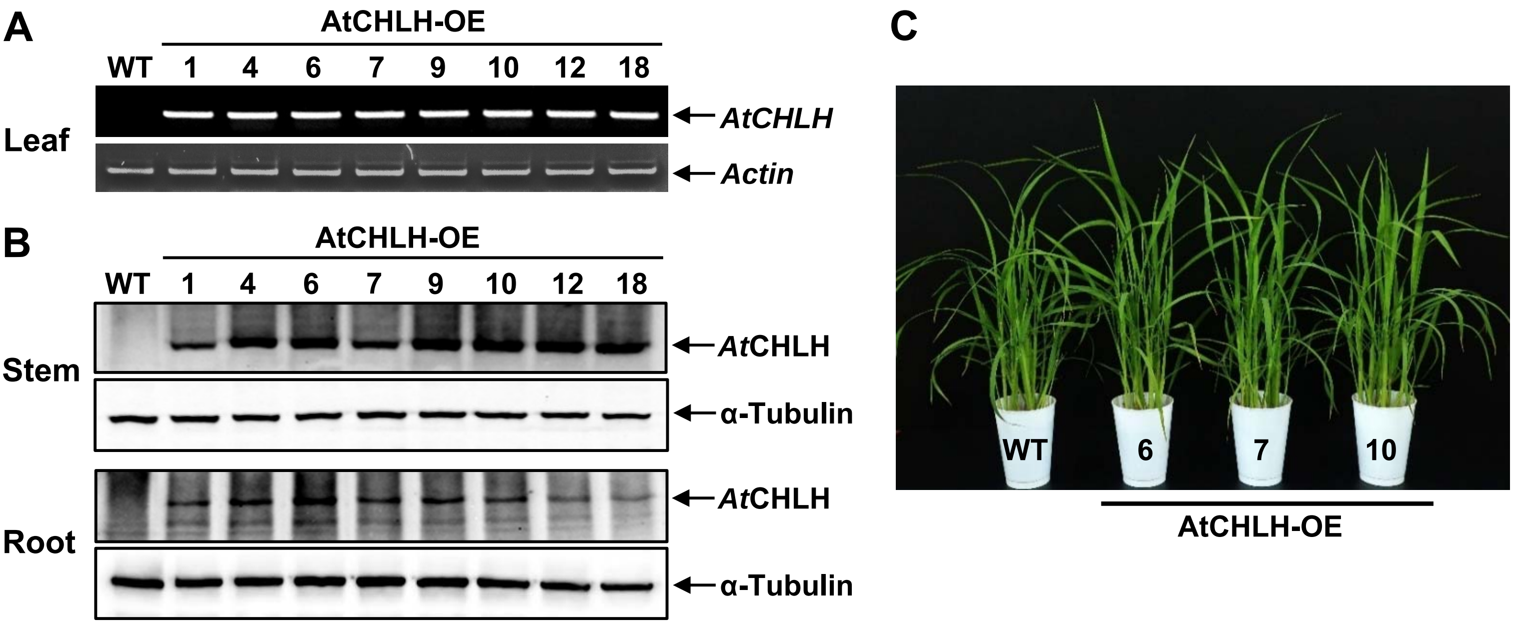

Supplementary Figure 1. Analysis of the transgene and phenotypes in WT and transgenic rice plants overexpressing *AtCHLH* grown under optimal conditions.

A, PCR analysis of *AtCHLH* gene from genomic DNA in leaves of transgenic lines. *Actin* gene was used as an internal control. **B**, Immunoblot of *At*CHLH protein using antibody against *At*CHLH in stems and roots of transgenic lines. *Alpha*-tubulin was used as a loading control. **C**, Phenotypes of WT and transgenic rice plants grown in the greenhouse under optimal conditions for 4 weeks. WT, non-transgenic wild type; AtCHLH-OE1–18, transgenic lines overexpressing the *AtCHLH* gene.

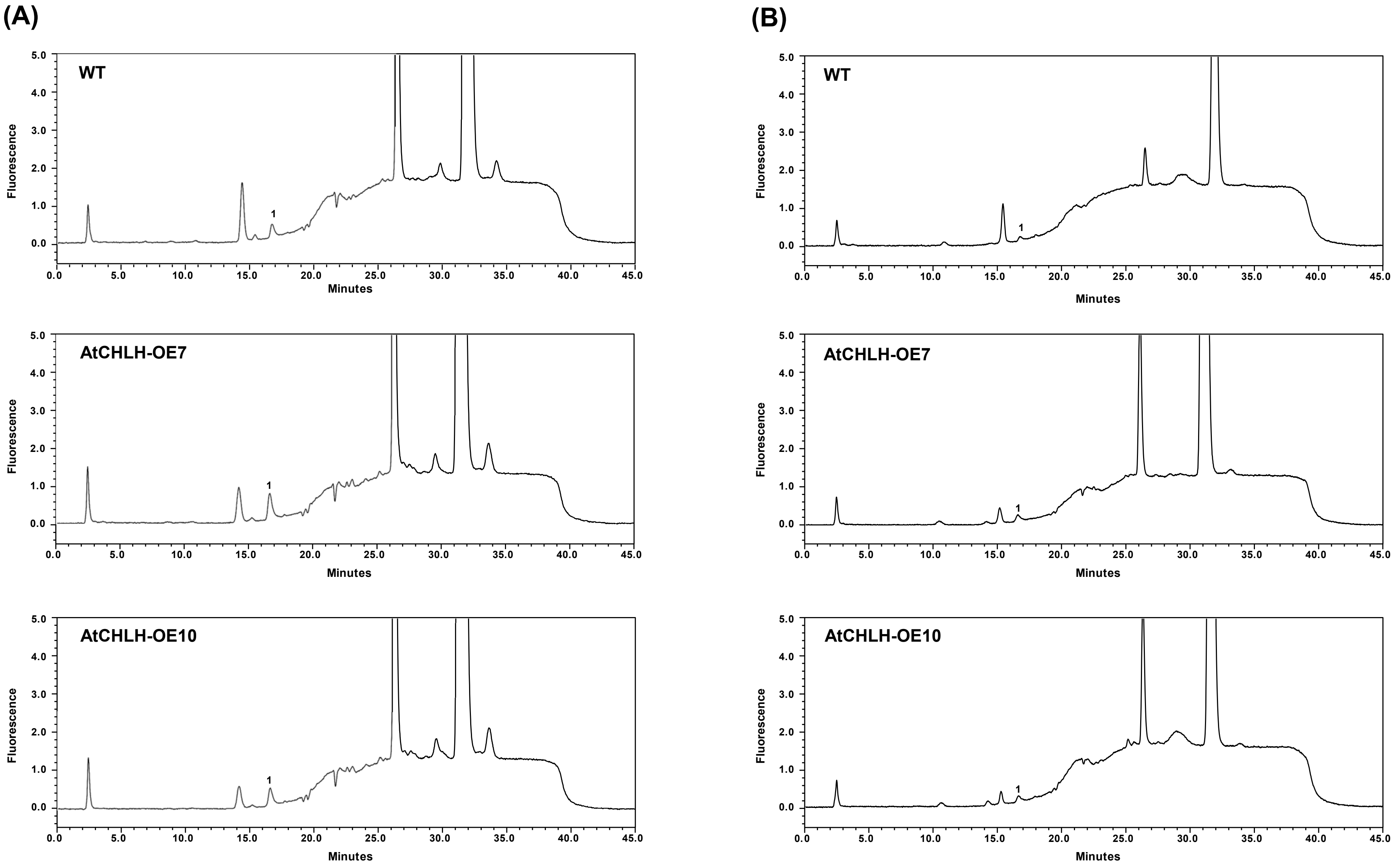

Supplementary Figure 2. HPLC chromatograms of Proto IX in WT and transgenic plants expressing *AtCHLH* under Fe-sufficient (**A**) and Fe-deficient conditions (**B**). WT and transgenic rice seedlings were transferred to half-strength Hoagland solution without or with 50 μM Fe-EDTA for 7 days. AtCHLH-OE7 and AtCHLH-OE10, transgenic lines overexpressing *AtCHLH*. 1, Proto IX.

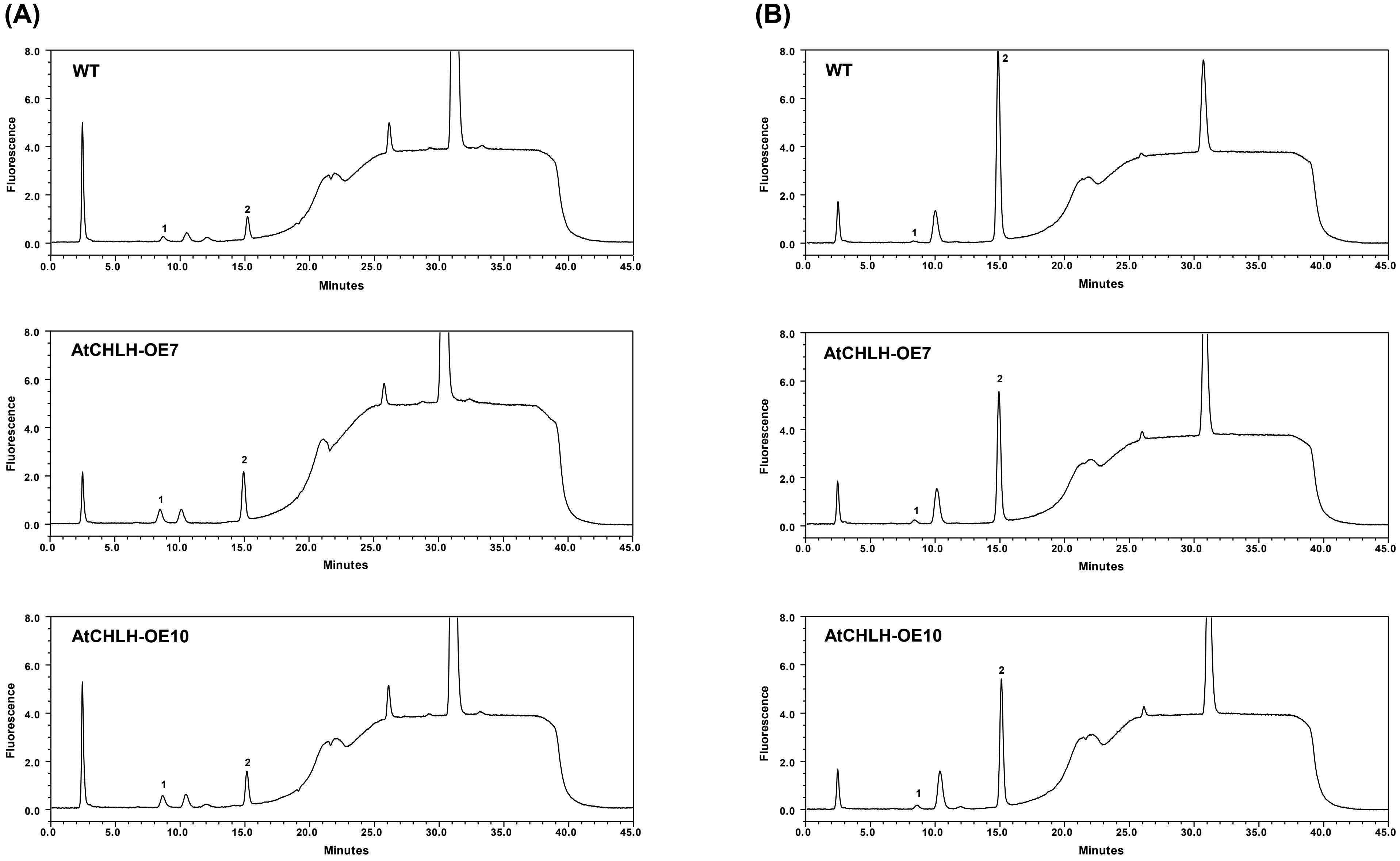

Supplementary Figure 3. HPLC chromatograms of Mg-porphyrins in WT and transgenic plants expressing *AtCHLH* under Fe-sufficient (**A**) and Fe-deficient conditions (**B**). WT and transgenic rice seedlings were transferred to half-strength Hoagland solution without or with 50 μM Fe-EDTA for 7 days. AtCHLH-OE7 and AtCHLH-OE10, transgenic lines overexpressing *AtCHLH*. 1, Mg-Proto IX; 2, Mg-Proto IX ME.

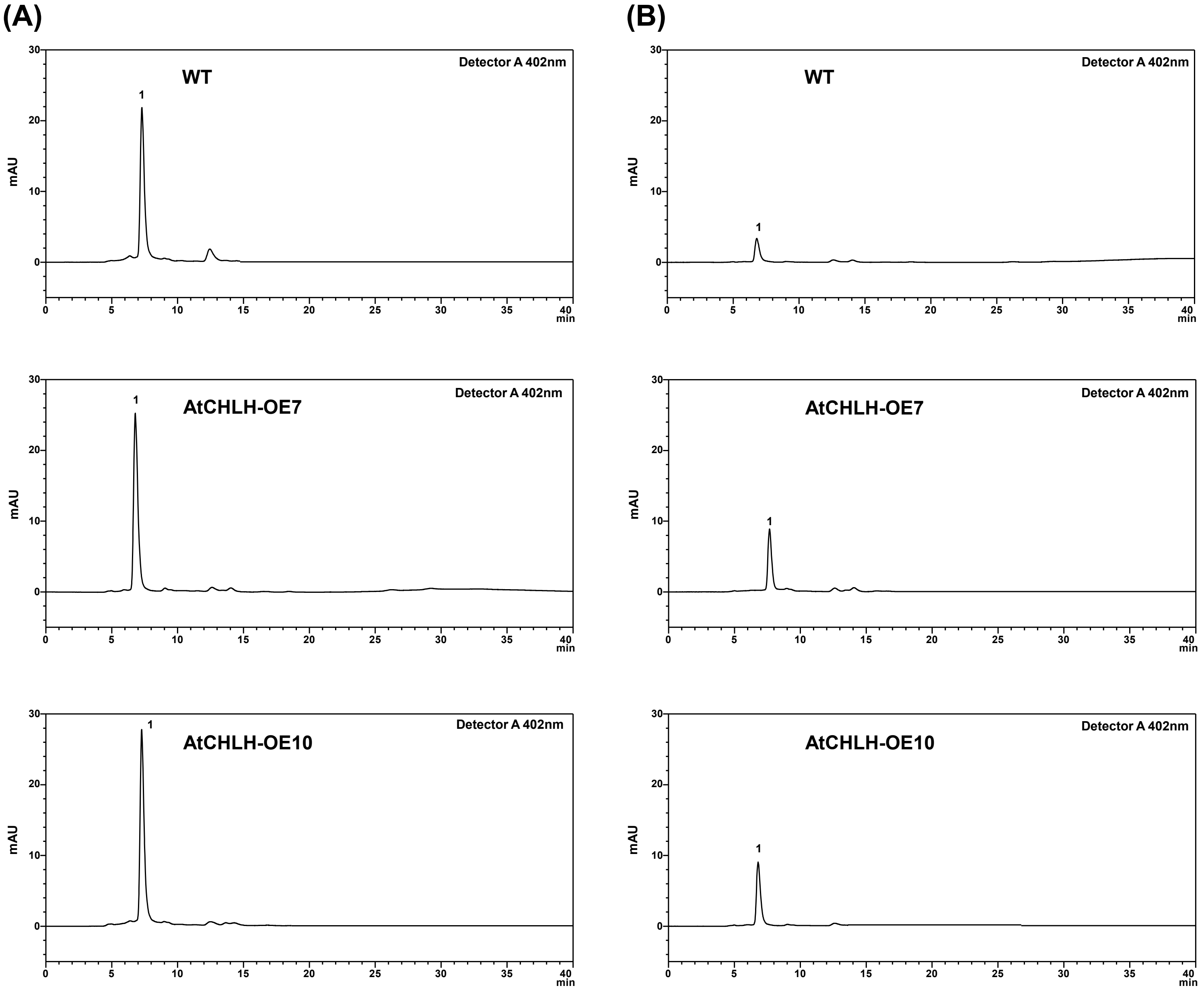

Supplementary Figure 4. HPLC chromatograms of heme in WT and transgenic plants expressing *AtCHLH* under Fe-sufficient (**A**) and Fe-deficient conditions (**B**). WT and transgenic rice seedlings were transferred to half-strength Hoagland solution without or with 50 μM Fe-EDTA for 7 days. AtCHLH-OE7 and AtCHLH-OE10, transgenic lines overexpressing *AtCHLH*. 1, Heme.

```
AtCHLH      1 MASLVYSPFTLSTSKAEHLSSLTNSTKHSFLRKKHRSTKPA----KSFFKVKSAVSGNGL
OsCHLH      1 MSSLVSTPFTTATGVQKKLGAPV--PLHSFLLSRRQPAAGAGRGRAAAAAIRCAVAGNGL
              * ***  ***  *     *        ****         *            ** ****

AtCHLH     57 FTQTNPEVRRIVPIKRD----NVPTVKIVYVVLEAQYQSSLSEAVQSLNKTS-RFASYEV
OsCHLH     59 FTQTKPEVRRVVPPEGDASRRGVPRVKVVYVVLEAQYQSSVTAAVRELNADPRRAAGFEV
              **** ***** **   *     ** ** ************   **  **    * *  **

AtCHLH    112 VGYLVEELRDKNTYNNFCEDLKDANIFIGSLIFVEELAIKVKDAVEKERDRMDAVLVFPS
OsCHLH    119 VGYLVEELRDEETYKTFCADLADANVFIGSLIFVEELALKVKDAVEKERDRMDAVLVFPS
              **********  **  ** ** *** ************ *********************

AtCHLH    172 MPEVMRLNKLGSFSMSQLGQSKSPFFQLFKRKKQGSAGFADSMLKLVRTLPKVLKYLPSD
OsCHLH    179 MPEVMRLNKLGSFSMSQLGQSKSPFFQLFKRKKN-SGGFADSMLKLVRTLPKVLKYLPSD
              *********************************  * ***********************

AtCHLH    232 KAQDARLYILSLQFWLGGSPDNLQNFVKMISGSYVPALKGVKIEYSDPVLFLDTGIWHPL
OsCHLH    238 KAQDARLYILSLQFWLGGSPDNLQNFLKMIAVSYVPALKGADIKYDDPVLFLDAGIWHPL
              ************************** ***  ********  * * ******* ******

AtCHLH    292 APTMYDDVKEYWNWYDTRRDTNDSLKRKDATVVGLVLQRSHIVTGDDSHYVAVIMELEAR
OsCHLH    298 APTMYDDVKEYLNWYGTRRDTNDKLKDPNAPVIGLVLQRSHIVTGDDGHYVAVIMELEAK
              *********** *** ******* **   * * ************** ***********

AtCHLH    352 GAKVVPIFAGGLDFSGPVEKYFVDPVSKQPIVNSAVSLTGFALVGGPARQDHPRAIEALK
OsCHLH    358 GAKVIPIFAGGLDFSGPTQRYLVDPITGKPFVNAVVSLTGFALVGGPARQDHPKAIAALQ
              **** ************   * ***    * **  ****************** ** **

AtCHLH    412 KLDVPYLVAVPLVFQTTEEWLNSTLGLHPIQVALQVALPELDGAMEPIVFAGRDPRTGKS
OsCHLH    418 KLDVPYIVALPLVFQTTEEWLNSTLGLHPIQVALQVALPELDGGMEPIVFAGRDPRTGKS
              ****** ** ********************************* ****************

AtCHLH    472 HALHKRVEQLCIRAIRWGELKRKTKAEKKLAITVFSFPPDKGNVGTAAYLNVFASIFSVL
OsCHLH    478 HALHKRVEQLCTRAIRWAELKRKTKEEKKLAITVFSFPPDKGNVGTAAYLNVFNSIYSVL
              *********** ***** ******* *************************** ** ***

AtCHLH    532 RDLKRDGYNVEGLPENAETLIEEIIHDKEAQFSSPNLNVAYKMGVREYQDLTPYANALEE
OsCHLH    538 QDLKKDGYNVEGLPDTAEALIEEVIHDKEAQFNSPNLNVAYRMNVREYQSLTSYASLLEE
               *** *********  ** **** ******** ******** * ***** ** **  ***

AtCHLH    592 NWGKPPGNLNSDGENLLVYGKAYGNVFIGVQPTFGYEGDPMRLLFSKSASPHHGFAAYYS
OsCHLH    598 NWGKPPGNLNSDGENLLVYGKQYGNVFIGVQPTFGYEGDPMRLLFSKSASPHHGFAAYYT
              ********************* *************************************

AtCHLH    652 YVEKIFKADAVLHFGTHGSLEFMPGKQVGMSDACFPDSLIGNIPNVYYYAANNPSEATIA
OsCHLH    658 FVEKIFQADAVLHFGTHGSLEFMPGKQVGMSDACYPDSLIGNIPNIYYYAANNPSEATVA
               ***** *************************** ********** ************ *

AtCHLH    712 KRRSYANTISYLTPPAENAGLYKGLKQLSELISSYQSLKDTGRGPQIVSSIISTAKQCNL
OsCHLH    718 KRRSYANTISYLTPPAENAGLYKGLKQLSELISSYQSLKDTGRGPQIVSSIISTAKQCNL
              ************************************************************
```

```
AtCHLH       772 DKDVDLPDEGLELSPKDRDSVVGKVYSKIMEIESRLLPCGLHVIGEPPSAMEAVATLVNI
OsCHLH       778 DKDVPLPEEGVELPPNERDLIVGKVYAKIMEIESRLLPCGLHVIGEPPSAIEAVATLVNI
                 **** ** ** ** *  **  ***** *********************** *********

AtCHLH       832 AALDRPEDEISALPSILAECVGREIEDVYRGSDKGILSDVELLKEITDASRGAVSAFVEK
OsCHLH       838 ASLDRPEDEIYSLPNILAQTVGRNIEDVYRGSDKGILADVELLRQITEASRGAITTFVER
                 * ********  ** ***  *** ************* *****  ** *****   ***

AtCHLH       892 TTNSKGQVVDVSDKLTSLLGFGINEPWVEYLSNTKFYRANRDKLRTVFGFLGECLKLVVM
OsCHLH       898 TTNNKGQVVDVTNKLSTMLGFGLSEPWVQHLSKTKFIRADREKLRTLFTFLGECLKLIVA
                 *** *******  **   ****  ****  ** *** ** * **** * ******** *

AtCHLH       952 DNELGSLMQALEGKYVEPGPGGDPIRNPKVLPTGKNIHALDPQAIPTTAAMASAKIVVER
OsCHLH       958 DNELGSLKLALEGSYVEPGPGGDPIRNPKVLPTGKNIHALDPQAIPTTAALKSAKIIVDR
                 *******  **** ************************************  **** * *

AtCHLH      1012 LVERQKLENEGKYPETIALVLWGTDNIKTYGESLGQVLWMIGVRPIADTFGRVNRVEPVS
OsCHLH      1018 LLERQKVDNGGKYPETIALVLWGTDNIKTYGESLAQVLWMIGVRPVADTFGRVNRVEPVS
                 * ****  * ************************ ********** **************

AtCHLH      1072 LEELGRPRIDVVVNCSGVFRDLFINQMNLLDRAIKMVAELDEPVEQNFVRKHALEQAEAL
OsCHLH      1078 LEELGRPRIDVVINCSGVFRDLFINQMNLLDRAVKMVAELDEPEEMNYVRKHAQEQAREL
                 ************ ******************** ********* * * ***** ***  *

AtCHLH      1132 GIDIREAATRVFSNASGSYSANISLAVENSSWNDEKQLQDMYLSRKSFAFDSDAPGAGMA
OsCHLH      1138 GVSLREAATRVFSNASGSYSSNVNLAVENASWTDEKQLQDMYLSRKSFAFDCDAPGAGMR
                 *   **************** *  ***** ** ****************** *******

AtCHLH      1192 EKKQVFEMALSTAEVTFQNLDSSEISLTDVSHYFDSDPTNLVQSLRKDKKKPSSYIADTT
OsCHLH      1198 EQRKTFELALATADATFQNLDSSEISLTDVSHYFDSDPTKLVQGLRKDGRAPSSYIADTT
                 *    ** ** **  ************************ *** ****   *********

AtCHLH      1252 TANAQVRTLSETVRLDARTKLLNPKWYEGMMSSGYEGVREIEKRLSNTVGWSATSGQVDN
OsCHLH      1258 TANAQVRTLSETVRLDARTKLLNPKWYEGMMKSGYEGVREIEKRLTNTVGWSATSGQVDN
                 ******************************* ************* **************

AtCHLH      1312 WVYEEANSTFIQDEEMLNRLMNTNPNSFRKMLQTFLEANGRGYWDTSAENIEKLKELYSQ
OsCHLH      1318 WVYEEANATFIEDEAMRKRLMDTNPNSFRKLVQTFLEASGRGYWETSEENLEKLRELYSE
                 ******* *** ** *  *** ********  ****** ***** ** ** *** ****

AtCHLH      1372 VEDKIEGIDR
OsCHLH      1378 VEDKIEGIDR
                 **********
```

Supplementary Figure 5. Amino acid sequence alignment of *At*CHLH and *Os*CHLH. *At*CHLH protein has 81% identity with the *Os*CHLH. To detect *At*CHLH, the polyclonal antibody was raised in rabbits against the synthesized peptide antigen used for antibody production. For designing and synthesizing the peptide antigen, cross-reactivity of AtCHLH (Q9FNB0) with *Os*CHLH (Q10M50) was considered to minimize risk of the cross-reactivity. Highlighted sequence in gray color was used for the synthesized peptide antigen.

Supplemental Table S1. Primers used for RT-qPCR assays.

Gene	Primer sequences
HEMA1	F: GCTATGGGTGGTGTTCGACT<br>R: CGATCTTCTGGAGGCACTTC
GSA	F: CTCCGTGACTTGACGAAACA<br>R: GTAGGTTCCAGGCTCCATCA
ALAD	F: GTCCACCGTCTCCTTCTCC<br>R: TGTCAAGTCAAGAGGCCTGA
PPO1	F: ACAGTTCCTCATTGGCCATC<br>R: CCCATGAAATTTTTGCTGCT
CHLD	F: TGGGACAGCAAAGACAGTGA<br>R: AAGGCCAGGTTGAAACACAG
CHLH	F: GAGACGACGGTCACTACGTC<br>R: TCTGTCGTCTGGAACACGAG
CHLI	F: TGTGCTTCTGGATTCTGCTG<br>R: GCTGGAGCTTGTCTTGTTCC
PORB	F: GTGAATTGCCAGGTTTTCGT<br>R: GCAATTAGCAAAGCTGCACA
FC2	F: TTGGTGCTATGGCAGTTTCA<br>R: AGTGGAACAAAGGCAGGATG
HO1	F: AGCGCTAGCAGTAGCAGGAG<br>R: GCTCCTTCTCCCCTTCCTT
HO2	F: TGCTTGAAAGAAGCTGCAAA<br>R: ATTGTTGTGGCCTGGTGTTT
AtCHLH	F: GTTAAGGATGCGGTGGAGAA<br>R: GCCTCCAAGCCAAAACTGTA
IRT1	F: CGTCTTCTTCTTCTCCACCACGAC<br>R: GCAGCTGATGATCGAGTCTGACC
IRT2	F: CGTTCCACACGCGGGGCAGCAAG<br>R: CCCATCCCCTCGAACATCT

NAS1	F: GTCTAACAGCCGGACGATCGAAAGG<br>R: TTTCTCACTGTCATACACAGATGGC
NAS2	F: TGAGTGCGTGCATAGTAATCCTGGC<br>R: CAGACGGTCACAAACACCTCTTGC
NAAT1	F: TAAGAGGATAATTGATTTGCTTAC<br>R: CTGATCATTCCAATCCTAGTACAAT
YSL2	F: TCTGCTGGCTTCTTTGCATTTTCTG<br>R: ACCATGTCGAACTCAGCATCCAGGA
YSL15	F: AACATAAGGGGGACTGGTAC<br>R:TGATTACCGCAATGATGCTTAG
IRO2	F: GGAGTGCCCAGCATTTTGTG<br>R: GACGATCTCGGAGTGCAACA
IDEF1	F: ACTCCCACTCCCAGAGGTTT<br>R: GTGCCATGGACGGTGAAGTA
IDEF2	F: TGAAGCATTTCTGCTTGTACCT<br>R: TTCAAGATCTCTGCTCTGGAGAC
Actin	F: CTTCATAGGAATGGAAGCTGCGGGTA<br>R: CGACCACCTTGATCTTCATGCTGCTA

F indicates forward and R indicates reverse.
